# Unveiling the Mysteries of Dyslexia—Lessons Learned from the Prospective Jyväskylä Longitudinal Study of Dyslexia

**DOI:** 10.3390/brainsci11040427

**Published:** 2021-03-27

**Authors:** Kaisa Lohvansuu, Minna Torppa, Timo Ahonen, Kenneth Eklund, Jarmo A. Hämäläinen, Paavo H. T. Leppänen, Heikki Lyytinen

**Affiliations:** 1 Department of Psychology, University of Jyväskylä, P.O. Box 35, FI-40014 Jyväskylä, Finland; timo.p.s.ahonen@jyu.fi (T.A.); jarmo.a.hamalainen@jyu.fi (J.A.H.); paavo.ht.leppanen@jyu.fi (P.H.T.L.);; 2 Department of Teacher Education, University of Jyväskylä, P.O. Box 35, FI-40014 Jyväskylä, Finland; minna.p.torppa@jyu.fi; 3 Niilo Mäki Institute, University of Jyväskylä, P.O. Box 35, FI-40014 Jyväskylä, Finland; heikki.j.lyytinen@jyu.fi; 4 Faculty of Education and Psychology, University of Jyväskylä, P.O. Box 35, FI-40014 Jyväskylä, Finland; kenneth.m.eklund@jyu.fi

**Keywords:** brain event-related potentials (ERPs), language development, dyslexia, home literacy environment, intervention, longitudinal study, prospective family study, reading fluency, reading development, reading difficulties

## Abstract

This paper reviews the observations of the Jyväskylä Longitudinal Study of Dyslexia (JLD). The JLD is a prospective family risk study in which the development of children with familial risk for dyslexia (N = 108) due to parental dyslexia and controls without dyslexia risk (N = 92) were followed from birth to adulthood. The JLD revealed that the likelihood of at-risk children performing poorly in reading and spelling tasks was fourfold compared to the controls. Auditory insensitivity of newborns observed during the first week of life using brain event-related potentials (ERPs) was shown to be the first precursor of dyslexia. ERPs measured at six months of age related to phoneme length identification differentiated the family risk group from the control group and predicted reading speed until the age of 14 years. Early oral language skills, phonological processing skills, rapid automatized naming, and letter knowledge differentiated the groups from ages 2.5–3.5 years onwards and predicted dyslexia and reading development, including reading comprehension, until adolescence. The home environment, a child’s interest in reading, and task avoidance were not different in the risk group but were found to be additional predictors of reading development. Based on the JLD findings, preventive and intervention methods utilizing the association learning approach have been developed.

## 1. Theoretical Premises

Developmental dyslexia is defined as an unexpected disability in learning to read [1,2]. Dyslexia manifests as severe and persistent difficulties in reading and writing, which are unexpected, as they appear despite adequate cognitive capacity and instructional or environmental opportunities. Yet, by its definition dyslexia is not caused by sensory impairment, lack of motivation, emotional disturbances, or any other possible extraneous factors. The neural basis of dyslexia has been investigated using brain research tools, e.g., [3,4,5,6]. These studies have found that, compared to the controls, differences in the function and organization of the core brain areas of the reading network in dyslexics and children at risk of dyslexia before reading instruction already have started.

The search term “dyslexia” yields more than 320,000 hits to research papers related to dyslexia in Google Scholar. As reading difficulties have a major impact on individuals’ possibilities to learn and participate in modern societies, it is understandable that vast resources are dedicated to the study of dyslexia to understand its mechanisms and to develop training tools. We believe that understanding dyslexia requires a developmental approach based on a prospective longitudinal study of children with and without a familial risk of dyslexia. This is because genes play a role in the complex interaction with the environment in the development of spoken and written language skills, e.g., [7,8,9]. Therefore, developmental effects need to be studied from an early age along with an examination of the learning environment, proceeding from the development of spoken to written language. A prospective approach allows us to look for early precursors of dyslexia and examine changes over time to obtain insights into the interactions among the genetic, neural, cognitive, and behavioral levels of description.

Our contribution to finding answers to the mysteries of dyslexia is the Jyväskylä Longitudinal Study of Dyslexia (JLD) [10,11,12,13]. The study is based on a prospective family risk design, which is useful for identifying early precursors of heritable developmental disabilities. By following the development of children born into families in which one of the parents and at least one of that parent’s close relatives have the disability in question, we can learn about the early development of children with an increased risk of developing the same disability. When we compare the development of children with familial risk and their peers without the risk (controls) and conduct longitudinal prediction analyses, we are able to identify early risk, protective, and promotive factors, which can be useful in trying to prevent or mitigate the reading problems.

In the JLD, we have now followed the development of 108 children with familial risk from birth to over 20 years of age. Their development has been compared with 92 children who have no known family history of dyslexia. In this article, we attempt to cover the all of the JLD’s findings regarding reading development from birth to early adulthood. 

The main goals of the JLD were to (1) identify the precursors and predictors of dyslexia; (2) specify the developmental paths leading to reading difficulties; (3) examine the contribution of environmental factors associated with dyslexia; (4) examine the developmental problems co-occurring with dyslexia; and (5) develop a methodology for early assessment and intervention. In this article, we aim to summarize the most important results from the JLD project. To provide the most effective preventive support, the children at real risk of reading problems should be identified before those problems manifest in the context of reading acquisition. In addition, the identification of precursors and predictors can reveal underlying neural and cognitive differences between those who learn to read normally and those who are in need of prevention. Thus, the majority of our summary comprises findings related to early identification.

## 2. Description of the JLD

### 2.1. Participants and Procedure

**Figure 1:**
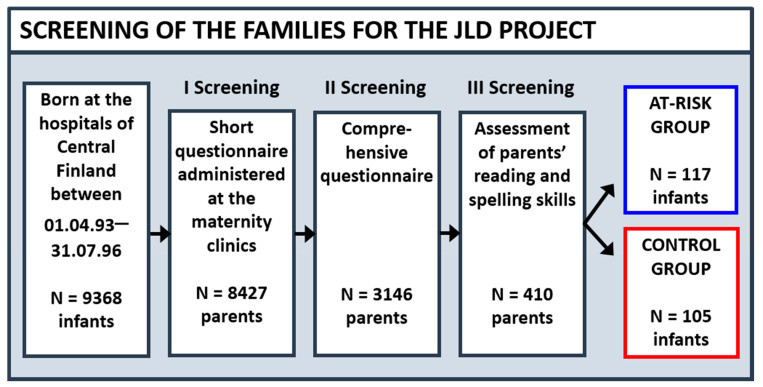
Screening of the families for the Jyväskylä Longitudinal Study of Dyslexia.

The participants of the JLD project were screened from among 9368 newborns born in the province of Central Finland between April 1993 and July 1996 (see Figure 1). The selection procedure included the following three stages: (1) a short parental questionnaire on the difficulties experienced by parents and their close relatives in learning to read and spell; (2) a detailed parental questionnaire on their own reading history and the persistence of their reading and spelling difficulties; and (3) an assessment of the parents’ reading, spelling, and related cognitive skills. Based on these, the children were classified into one of two groups: the group with family risk (FR) for dyslexia (the at-risk group) or the group without family risk for dyslexia (the control group). During the first year, some families from both groups decided to drop out of the longitudinal follow-up study, and therefore the final numbers of participants were 108 for the at-risk group and 92 for the control group.

To be included in the FR group, at least one parent of the child had to perform poorly in oral text reading or spelling and in phonological and orthographic processing tasks. Another criterion was that a parent had to report the onset of literacy problems in the early school years. In addition, a report of at least one first-degree relative of the index parent with similar difficulties was required. To be included in the control group, both parents of the child were required to report no family history of dyslexia and to achieve a z-score above -1 in all reading and spelling tasks. The IQ scores of the parents in both groups had to be equal to or higher than 80 (for full details on the recruitment procedure, see [10].

**Figure 2:**
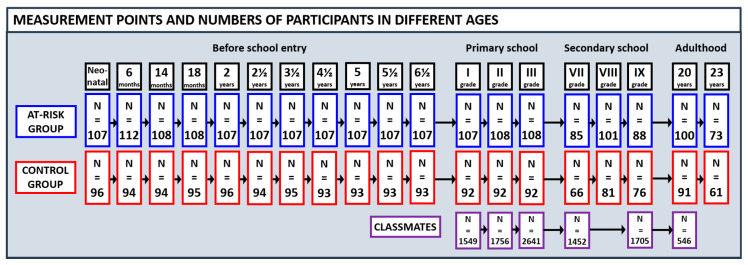
Measurement points and numbers of participants at each age phase.

All the children were native Finnish speakers and attended regular classroom education. The dropout rate was very low in the assessments before school age and during the first school years up to the end of Grade 3 (at the age of 10 years); until that time, almost all 200 children participated in all the assessments. In adolescence, in Grades 7 to 9, 151–182 children continued to participate in the study. Figure 2 shows the numbers of participants at each age phase.

### 2.2. Measures

A broad assessment battery covering brain measures, key cognitive skills, and environmental characteristics possibly predicting children’s later reading and spelling skills were employed. Measures for each measurement point were selected based on the theoretical and empirical knowledge available at the time of each assessment phase. Validated measures were utilized whenever possible, but in the cases where no such measures were available in Finnish, new measures were developed and validated within the project. The assessment battery consisted of tests, EEG/ERP measurements and observations in the laboratory, assessments in the classrooms, and questionnaires for parents, teachers, and children. A complete list of the assessments utilized at each measurement point of the JLD project are presented in Supplementary Materials (Table S1). For further details, see the original publications on the topic.

Cognitive, linguistic, and motor skills: Children’s cognitive development was assessed in the domains of oral language, general cognitive skills, motor skills, phonological skills, memory, rapid automatized naming, and orthographic skills, as appropriate at different ages from the age of one year onward. Besides the assessments, data related, for example, to language and motor development, were collected through parental questionnaires and diaries from birth onward.

Learning environment: Environmental characteristics at home, daycare, and school were assessed via yearly parental questionnaires, observations, and teacher questionnaires. 

Behavior and temperament: Questionnaires completed by parents and teachers included evaluations concerning the child’s temperament, attention, internalizing problems, and externalizing problems.

Child’s interest in reading and task avoidance: Child’s interest in reading and task avoidance were assessed by parental questionnaires in childhood (from 2–10 years of age), while participants’ self-reports were utilized in adolescence (from 13 to 15 years of age).

EEG measurements: The EEG measurements of the JLD project were conducted for newborns, at 6 months, in kindergarten at the age of 6.5 years, and in Grade 3 at the age of approximately 9 years. Most EEG (more specifically, brain event-related potential, ERP) measurements focused on speech perception and some of them on more basic auditory processes. The focus was mostly on processing of phonemic length (duration), as this feature, called quantity, is an important semantically distinguishing feature in the Finnish language. Thus, for example, words like tuli (fire), tuuli (wind), and tulli (customs) differ only in terms of the quantity perception of one phoneme (either /u/ or /l/). When learning to read and write, these variations in phoneme length are often the most difficult, even for children without any problems in reading acquisition. The last spelling accuracy errors children make usually relate to phonemic length-related details. 

Auditory brain processing of speech stimuli involving consonant length variation was examined at 6 months, 6.5 years, and 9 years [14,15,16]. This involved using a pseudoword /ata/ and its longer versions /at:a/ in an oddball design. The stimulus was a naturally produced short version of the pseudoword, including a single stop consonant /t/, and the longer versions were created by extending the silent period to create longer variants of the consonant, the longest version including a geminate /t:/ [17]. Processing of vowel length was examined 1–7 days after the birth and at 6 months (the latter assessments were conducted in about half of the JLD children) [18,19]. This ERP measurement involved the syllables /ka:/ and /ka/, which were also presented in an oddball experiment. At 1–7 days after birth, speech perception was also studied using an experiment presenting different syllables (/ba:/, /da:/, /ga:/, /pa:/, /ta:/, /ka:/, and /ka/) with equal probability to examine basic obligatory brain responses without the need for memory-based comparison processes, in contrast to the brain responses measured in the oddball experiments [20]. This design was also used in Grade 3 for a sub-sample of the JLD children.

Basic auditory processing was measured in newborns, at 6.5 years, and at 9 years utilizing non-linguistic stimuli. During the first week after birth, detection of pitch change (1000 Hz vs. 1100 Hz sinusoidal sounds) was studied using an oddball design [21,22]. At 6.5 years and 9 years, more complex, non-speech sounds were used to assess basic processing of variations in duration elements of auditory stimuli [15,23]. The purpose here was to use them as comparison points to the speech stimuli in order to investigate how speech specific the processing differences between children at risk for dyslexia and the controls were [15], and partly, to answer specific hypotheses on the processing of amplitude envelopes and rapidly presented stimuli [23]. The technical details of the EEG measurements at each age are presented in Supplementary Material (Table S2).

## 3. Key Findings of the JLD

The results of the JLD project are reviewed below, starting from the brain-related observations and ending with the preventive intervention tool developed to support children’s reading acquisition. In the following sections, we describe our results on children’s early brain level and cognitive skill associations, precursors, and predictors of dyslexia. We also illustrate how the environmental and motivational factors correlate with the development of skills, which have been followed over a long time period and are, by now, reported until adolescence, covering reading fluency and reading comprehension.

### 3.1. The Criteria for Dyslexia

The Finnish educational system does not require a dyslexia diagnosis for children to be eligible to receive special education or other remedial support for literacy difficulties. Therefore, dyslexia is relatively rarely officially diagnosed in Finland, and for that reason, the following dyslexia identification criteria were used in the JLD studies. The classification of children with and without dyslexia was done for the first time at the end of Grade 2 at the age of 8–9 years [24]. The choice of this time point was based on the national core curriculum for basic education [25], according to which basic reading techniques should be learned by the end of Grade 2. To be classified with dyslexia, the child’s skills had to be at or below the 10th percentile of the performance of the control group (a) in at least three out of the four reading/spelling accuracy measures; (b) in at least three out of the four reading fluency measures; or (c) in two accuracy and two fluency measures. Using these criteria, 38 children (35.8%) with family risk and nine children (9.8%) without family risk were identified as having dyslexia.

In Grade 8, we could no longer use the same criteria as in Grade 2 because, for Finnish readers, the reading accuracy measures show the ceiling effect after the early grades. This is typical in consistent orthographies, where dyslexia is manifested as extremely slow and laborious decoding despite relatively few decoding errors [26,27,28]. Therefore, in Grade 8 we identified dyslexia based on reading fluency in word list reading, text reading, and pseudoword text reading [29,30]. Similarly to the Grade 2 dyslexia definition, performance below the 10th percentile (of the distribution of the controls) in at least two out of these three measures was considered to be an indication of difficulties in reading fluency. In Grade 8, approximately the same percentage of participants were identified with dyslexia as in Grade 2, and in the FR group poor reading and spelling skills were fourfold compared to the control group (approximately 10% versus 40%).

We also examined the stability of the diagnosis across time [30]. Note that in the study in which the stability of dyslexia was examined from Grade 2 to 8, the same reading fluency criterion was used in both grades for consistency across time. Interestingly, moderate instability of dyslexia status across the two time points was found, which was not fully due to the random changes in cut-offs [30]. Of the 55 children who were identified as having dyslexia at either of the two evaluation time points, three subgroups with different developmental trajectories were distinguished. The Persistent group fulfilled the criteria for dyslexia in both Grade 2 and Grade 8, and it consisted of the majority (40%, 10 girls/12 boys) of the participants with dyslexia. The Late-Emerging subgroup (33%, 4 girls/14 boys) showed no dyslexia in Grade 2 but fulfilled the criteria of dyslexia in Grade 8. The third subgroup, the Resolving (27%, 12 girls/3 boys), included the participants who had dyslexia in Grade 2 but not in Grade 8.

### 3.2. Precursors of Dyslexia

#### 3.2.1. Brain-Level Findings

A widely agreed causal risk factor for dyslexia is phonological deficit, suggested to originate from poor auditory and speech perception [31,32,33]. Phonological deficit in dyslexia is thought to hinder the learning of fluent and automatic decoding of grapheme–phoneme correspondences and to result in effortful reading and spelling. At a neural level, a speech perception deficit is considered a risk factor for dyslexia (for reviews, see [31,34,35,36,37]). Brain studies suggest that a phonological deficit may be caused by inaccurate, inflexible, or otherwise inadequate phonological representations in the brain caused by lower-level auditory or speech-processing problems [32,38,39,40]. In other words, when the brain is unable to adequately discriminate all the features of speech or categorize speech sounds, cf. [41], the representations in the brain cannot develop well enough, and this causes problems on higher levels, such as phonological processing and reading (for a review, see [42]). 

To explain the causal chain from neural-level auditory and speech processing deficits to reading problems via a phonological deficit, both top-down and bottom-up theories have been developed. Nevertheless, there is no clear consensus regarding the association between speech processing and reading deficit or dyslexia (for a review, see [43]). As an example of bottom-up theories, it has been proposed that the perception of different rates of speech (involving slow modulations in speech related to syllable processing and faster modulations related to phoneme processing) have an impact on phonological processing, the ability to manipulate speech sound components, and reading skills, e.g., [44,45]. Further, a study that investigated speech processing with functional magnetic resonance imaging (fMRI) found deficits in access to phonetic representations, although no deficits in the cortical brain processing of speech sounds were found [46]. However, this result could reflect an end state in adulthood, while during childhood the phonetic representations are also altered in dyslexia [47]. 

Top-down theories include suggestions that the deficits in higher linguistic processes at sub-lexical and lexical levels cause phonological problems and that lower-level problems do co-occur, but they are not considered a cause for dyslexia [48,49]. Brain-level observations have also revealed that learning to read enhances phonological activation to speech in the planum temporale, which is involved in auditory processing [50]. It could also be possible that, at the brain level, the abnormalities in the reading network in dyslexics may be the consequence of different reading experiences compared to normal readers. Indeed, it is well known that brain structures and particularly the functioning of brain regions change with experience and environmental input, e.g., [51,52,53]. At the time when the JLD project was started, there were no ERP studies, including very young infants at familial risk of dyslexia. Today, although substantial evidence for neurobiological risk factors for dyslexia have been reported (for reviews, see [5,54,55,56]), the results still mainly rely on measurements in adults and older children, which complicates the identification of possible causes and consequences. Subsequently, following in the footsteps of the JLD project, other studies also reported early brain measures related to dyslexia risk [57,58,59,60].

To identify the fundamental causes of dyslexia, very early longitudinal follow-up is essential. The first indices that are possible to obtain are the brain responses that can be measured in infants right after the birth. Much earlier than the child is able to consciously react to stimuli (e.g., turn head, speak, or read), brain activations to auditory or speech stimuli can shed light on early development, showing the effect of genetic factors as purely as possible before extensive exposure to environmental factors shuffles the puzzle. With this in mind, in the JLD project brain research has been employed to measure brain event-related potentials (ERPs) in children a few days after birth, at 6 months, in kindergarten at 6.5 years, and in Grade 3 at 9 years, e.g., [14,15,16,18,22,36,61,62,63]. The results have shown that very early age auditory and speech perception is different in children at risk for dyslexia compared to control children with low or no risk for dyslexia. These very early precursors of dyslexia seem to be associated with reading problems not directly but through cognitive precursors, such as a phonological deficit or difficulties in rapid automatized naming or letter naming ability.

Throughout our longitudinal study, we have reported differentiated brain activation between infants or children with and without familial risk for dyslexia in response to auditory non-speech and speech stimuli in all ages in which the ERP measurements were conducted. During the first week after birth, brain responses to basic-level auditory and speech processing were investigated. To study basic auditory processing, the mismatch negativity (MMN) paradigm with tonal stimuli (frequently presented standard of 1000 Hz and rarely presented deviant of 1100 Hz) was utilized to reveal neuronal-level pitch-change detection [21,62]. The change-detection responses of at-risk infants who later had reading problems at school age as well as those of at-risk infants who later developed typical reading skills were compared with the responses of the control infants. The control group showed clear bilateral change-detection responses, but for both the at-risk groups the change-detection responses differed from the control group, being smaller in amplitude in the at-risk groups. Additionally, at the age of 1–7 days, the basic afferent stimulus-driven brain responses to speech sounds, including the syllables /ba/, /da/, and /ga/, were obtained [20,61,64]. The at-risk and control infants differed in their response to the stimulus /ga/ in the right hemisphere: the responses of the at-risk infants were more positive and longer lasting in comparison to the control infants, suggesting an atypical hemispheric response pattern and atypically slow return back to the baseline, likely due to less efficient speech sound processing.

In Finnish, categorization based on phonemic length (i.e., speech sound duration of vowels and consonants) is an essential semantic feature, which alters the meaning of a word. Therefore, it is essential to be able to discriminate between the short and long versions with precision, enabling accurate categorization of short and long phonemes (singleton and geminate consonants in the case of consonants). In newborn and 6-month-old infants, brain responses to differing vowel durations (long standard /ka:/ vs. short deviant /ka/) were measured, and the brain activation was found to be different in the at-risk and control groups [1,18]. The brain responses to the deviant stimulus in newborn at-risk infants were, similarly to the basic responses to /ga/ syllables (see above), atypically large in several scalp areas, especially in the right hemisphere, compared to the control infants. This suggests that occasional shortening of vowel duration induces enhanced brain activation in the right hemisphere in at-risk newborns. 

In addition to differences between the at-risk and control infant groups, the infant brain responses to both speech and non-speech stimuli were associated with later language-related measures [22,36,61,63,64]. The ERPs to tones were associated with phonological processing at 3.5 years, letter knowledge at 5 years, and speech perception, spelling, reading speed, and reading accuracy at 8 years in Grade 2 [22]. The ERPs in the right hemisphere to the syllable /ga/, showing group differences in the newborns, were found to be associated with receptive language skills in both groups at 2.5 years, while the responses of the left hemisphere were associated with verbal memory at the age of 5 years [61]. Later, these same responses were shown to be predictive of pre-reading skills, including phonological skills, rapid naming, and letter knowledge at the age of 6.5 years, in children with and without familial risk for dyslexia [62]. These results indicated a link between very early basic processing of non-speech and speech sounds and reading-related cognitive skills and reading itself.

At six months, ERPs to speech sounds were also measured using pseudowords involving singleton and geminate consonants. In the study of Leppänen et al. [14], a pseudoword /ata/ with three phonemic length variants were used as stimuli (see above under Section 2.2). The results showed that the basic brain activation of at-risk infants in response to regularly repeated pseudowords as well as the brain responses reflecting change detection of phonemic length was significantly different from the brain activation of the control infants. More recently, revisiting the same data after infants’ reading outcomes were measured several times when they were of school age, Lohvansuu et al. [63] found that the responses measured at six months to the frequently repeated pseudoword /ata/ were strongly associated with reading speed at the ages of 8, 10, and 14 years in the at-risk group. Correlations were also found with phonological skills, letter knowledge, and rapid automatized naming at preschool age. It is noteworthy that brain activation explained over 40% of the reading speed score at 14 years, and it even improved the prediction of reading speed beyond the preschool measures of phonology, letter naming, and verbal short-term memory. The association of the speech perception measure of infants to reading speed at adolescence was found to be mediated by rapid naming speed. The finding that brain indices can improve the explanatory power of later reading measures on top of cognitive skill measures is in line with the regression analysis conducted by Maurer et al. [65] of kindergarten-age children, who were tested again at school age. 

At the ages of 6.5 and 9 years, ERP measurements were performed using the same pseudowords [15,16]. The results showed persistent brain response differences at preschool age and school age; the measured ERPs were atypical in children with reading difficulties and associated with reading scores measured at school age. Together, these studies indicate atypical brain responses to auditory and speech stimuli in at-risk children who later become dyslexics from birth to school age. Furthermore, these atypical brain responses are predictive of later language and reading skills [15,16,22,61,62,63]. The findings of the ERP studies of the JLD project reviewed above are in line with those of other longitudinal studies, which have shown differences in brain function between infants with and without a familial risk of dyslexia and developmental language disorder (DLD) and predictive value for later language related skills e.g., [57,58,59,60,66,67].

#### 3.2.2. Language-Related Cognitive Predictors before School Age

One of the key aims of JLD was to identify early cognitive skills that would be early markers of subsequent difficulties in reading and spelling development. Therefore, the early language-related cognitive skills of children who were later identified to have dyslexia were compared with those of children with later typical reading skills either with or without a familial risk of dyslexia. Regarding predictive analyses of reading development and dyslexia, our early papers focused on the skills prior to school entry or in Grade 1, e.g., [68,69]. These studies suggested that letter knowledge is a particularly important early predictor of early decoding but also that letter knowledge itself is predicted by early vocabulary and development of phonological awareness.

**Figure 3:**
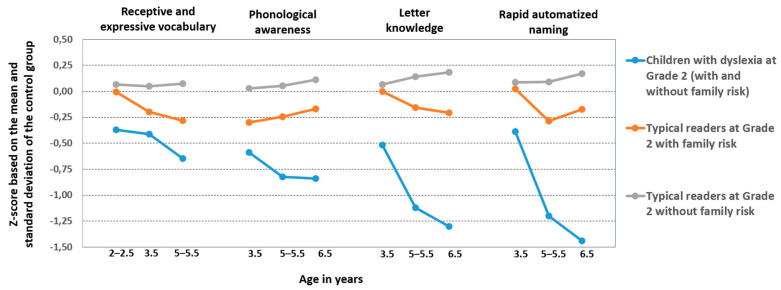
Group means of the key cognitive predictors of reading skills.

Our findings on the early cognitive markers of dyslexia in Grade 2 showed consistent differences when compared to typical readers without family risk in receptive and expressive language skills at 2–5.5 years and phonological sensitivity, letter knowledge, and rapid automatized naming at 3.5–5.5 years [70] (see Figure 3). Effect sizes were moderate, except for letter knowledge and rapid automatized naming at 5.5 years, where the effect sizes were large. In addition, these children differed from typical readers with family risk with a moderate effect size in expressive language skills at 2–2.5 years and letter knowledge and rapid naming at 3.5 and 5.5 years. In contrast to the findings in less transparent orthographies, e.g., [71,72,73,74,75], no differences were found between at-risk children without later dyslexia at school age and control children without dyslexia in these early cognitive skills, which gives only weak support to the idea of the continuous nature of vulnerability to reading difficulties.

The importance of letter knowledge as a predictor of early reading skills was repeated in the studies predicting Grade 2 dyslexia [24] as well as those using the latent factors of reading and spelling accuracy and reading fluency [76]. However, these studies also suggested an important role of rapid automatized naming (RAN) and phonological awareness. In terms of early prediction of children’s Grade 2 dyslexia, letter knowledge, rapid automatized naming, and phonological awareness assessed at the ages of 3.5, 4.5, and 5.5 years were the strongest predictors [24,76]. It was noteworthy, however, that, despite the inclusion of a broad battery of predictors (phonological awareness, rapid automatized naming, short-term memory, expressive vocabulary, pseudoword repetition, and letter knowledge), family risk remained an additional significant predictor of children’s dyslexia and reading skills over and above children’s skills. The three cognitive predictors (letter knowledge, rapid automatized naming, and phonological awareness) together with family risk explained 32–35% of the dyslexia status and effectively identified the individuals at risk for dyslexia years before school entry. Conversely, good early cognitive skills decreased the risk for dyslexia substantially among at-risk children. This suggests that the same cognitive predictors may serve not only as predictors but also as protective factors against dyslexia.

It is noteworthy that in the transparent orthography of Finnish, the effect of a deficient phonological awareness seems to be restricted to the very early phase of reading acquisition, after which it mainly affects spelling—and only in children with family risk [77]. More specifically, poor spelling accuracy was found to be associated with a compromised ability to discriminate phonemic length [78,79], which is in line with our brain research findings (see above) and highlights the importance of the phonological route in spelling of transparent orthography. In contrast, rapid automatized naming was related to reading speed throughout Grades 1 to 3 [77] and also characterized children with late-emerging dyslexia; that is, the group of children who were not identified as having dyslexia in Grade 2 but showed reading fluency deficiency in Grade 8 [30]. Interestingly, parents of these children also showed severe difficulties in rapid naming, suggesting a strong genetic liability of the skill.

Importantly, our findings suggest that individual risk for dyslexia is a combination of several risk factors [24], and therefore there are subgroups of children with different combinations and different developmental trajectories to early reading. In fact, our mixture modelling study inspecting preschool skills in seven domains (receptive vocabulary, expressive vocabulary, morphology, phonological awareness, rapid naming, letter knowledge, and verbal short-term memory) suggested three different routes through which children encountered problems in acquiring reading skills [11]. Children in the Dysfluent group were characterized by slow naming speed but had poor skills in early letter knowledge and vocabulary as well. The reading speed of this subgroup was the lowest compared to all other subgroups at the end of Grade 1. Children in the Declining group were characterized by an increasing lag in the typical development of all preschool skills examined, except naming speed. After Grade 1, these children showed clear deficiencies in both reading and spelling accuracy as well as in reading fluency. Children with family risk for dyslexia were overrepresented in the Dysfluent and Declining subgroups. Furthermore, family risk seemed to moderate the outcome of children in the Declining group; the children with a declining trajectory and familial risk performed worse in reading and spelling than the children without familial risk The third trajectory group, the Unexpected, showed proficient preschool skills in all other domains except in letter knowledge, which probably led these children to encounter problems in early reading acquisition [11]. This group included a similar amount of children with and without FR, and a relatively small number of children in this group (21%) entered having dyslexia at the end of Grade 2.

In a follow-up study, we examined the proportions of dyslexia in the abovementioned subgroups [80]. High proportions of children exhibited Grade 2 dyslexia in the Dysfluent (75%) and Declining (38%) subgroups. When the children with and without dyslexia within the subgroups were compared on multiple measures, several potential protective factors were found. The children who, despite their heightened early cognitive risk, avoided dyslexia in the Dysfluent and Declining subgroups had better Grade 1 phonological skills, and they were also evaluated as more task focused by their teachers. Children in the Unexpected subgroup having dyslexia in Grade 2 had poorer cognitive skills, read less alone during their free time, and were less task-focused according to their parents compared to children in the Unexpected group who did not have dyslexia [80]. Task-focused behavior also seemed to mediate the effect of early language skills on children’s spontaneous reading acquisition in this sample [81]; children who had good verbal skills at the age of 5 years showed a high level of task-focused behavior at the age of 6.5 years, which in turn predicted spontaneous reading acquisition.

#### 3.2.3. Environmental Factors 

The role of the home literacy environment (HLE) in children’s skill development has been one of the interests of JLD. Overall, studies on the HLE of children with familial risk for dyslexia remain scarce (see [32]), and the evidence is mixed. Some studies comparing HLE factors between children with and without family risk have found group differences, which suggests that family risk is at least partially mediated by the home environment [82,83,84], although other studies have not [9,38]. We have examined the key components (shared reading and teaching of print) of the influential HLE model [85,86] as well as parental education level, parent’s own reading activities and attitude, and children’s access to print (e.g., amount of books at home and library visits), and conducted observations on the book reading interactions between children and their mother, e.g., [68,69,87,88,89,90,91]. Our studies on the at-risk group differences in the HLE have generally reported no differences between families with and without family risk; an exception to this is the parent’s own reading activity [69,87,88,92]. As expected, parents with dyslexia tend to read less than parents without dyslexia. However, possibly due to the lack of differences in parental education (matched at the group level by design), they reported providing similar HLEs to their children as the parents without dyslexia. These findings suggest that, in the JLD sample, the risk of children’s poor reading development due to family risk (i.e., parental dyslexia) is not mediated by the HLE.

The JLD studies have found evidence that HLE factors, particularly shared reading and teaching of letters and reading at home, are associated with reading skills. In line with the HLE model [85,86], the JLD findings have suggested that meaning-focused activities, such as parent–child shared reading, are associated with oral language development, e.g., [69], and through oral language, contributes to the child’s reading comprehension skills later on [90]. Torppa et al. [89] also reported that the poor readers, who had difficulties in both reading fluency and reading comprehension development in Grades 1 and 2, had had fewer parent–child shared reading experiences than good readers prior to school entry. Moreover, in line with the HLE models, print-focused activities, such as teaching letters or reading, were predictive of development of letter knowledge at age 3.5–6.5 years in JLD [68,90]. According to our findings, the HLE factors that do not entail active interaction between parents and their children (number of books at home, library visits, or parent’s own reading activities) were not associated with children’s skill development. Furthermore, significant direct associations between HLE factors and children’s skills were found only for oral language and letter knowledge development, not for the other key cognitive predictors of reading development (RAN, phonological awareness).

Furthermore, it is possible that rich HLE is not only correlated with literacy development but also acts as a protective factor, closing the gap between children with and without family risk. There is some evidence in support of the protective role of HLE. Hamilton et al. [93] found that early storybook exposure indeed had a stronger predictive effect on children’s phonological awareness among children with family risk than among children with no risk. Furthermore, in the JLD sample, Torppa et al. [69,90] reported higher correlations between shared reading and vocabulary among children with family risk than among children without family risk. These findings suggest that shared reading with at-risk children may be particularly important and beneficial.

#### 3.2.4. Motivation and Attention

We have also examined the role of children’s interest in reading, inattention, and achievement strategies in skill development. Theoretically, children’s own reading interest can have an important effect on how much they engage in shared reading activities at home or in leisure time reading, which in turn can support skill development. Shared reading activities and active leisure time reading provide immense opportunities for practicing decoding and gaining exposure to orthographic patterns, e.g., [39], as well as gaining linguistic knowledge, such as a broader and deeper vocabulary, e.g., [94,95]. Previous studies have associated reading difficulties with attentional difficulties, e.g., [96,97], and task-avoidant behavior [98,99]. Inattention and task-avoidant achievement strategies may act in a similar manner to lower reading interest. If children have difficulty staying focused on tasks, they may practice reading less and thus have slower development and poorer reading performance. Conversely, both reading interest and strong attention and task focusing skills may act as a protective factor despite cognitive weaknesses.

Our findings regarding reading interest suggest, first, that the effect of family risk on children’s skills is not mediated by children’s interest, as family risk did not predict children’s reading interest. Children with and without family risk showed no differences in reading interest across time, from toddlers to adolescents, whether assessed using observational measures [88] or questionnaires [68,69,89,91]. This is interesting, as the parent’s own reading interest was lower in the family risk group than in the control group. Thus, the parents in the JLD study did not seem to pass on their reading interest directly to their children, at least not by adolescence. We did, however, identify correlations between shared reading and children’s interest [69,90,91] from age 2 onwards. Based on the JLD data, it is not possible to conclude which one, shared reading or child’s reading interest, is the chicken or the egg, but the association was already established at the age of 2 years, and shared reading was found to predict children’s reading interest until reaching school age. Upon reaching school age, children’s reading interest was associated with reading fluency and reading comprehension development [89,91]. At first, the associations were reciprocal but stronger from skill to interest, whereas in the long-run, when followed up to adolescence, there were significant predictive associations between reading interest and reading comprehension, and the association with reading fluency disappeared after Grade 3. Our findings regarding the JLD follow-up participants and their classmates in Grade 9 further supported this result, as the adolescents with reading comprehension difficulties reported various problems related to school motivation [100]. Importantly, the connections were not significantly different for the groups with and without family risk. It thus seems that, for adolescents with and without family risk, reading interest and active engagement in reading may support reading development. In addition, the supportive role of parent–child shared reading is not limited to oral language development but may also support the development of children’s reading interest.

The JLD studies on inattention have found the expected association with dyslexia. For example, Parhiala et al. [101] reported that children with dyslexia (identified in Grade 2) were more inattentive (based on parental reports) than typical readers years before school entry. In addition, an interesting gender difference was found. Boys with dyslexia had a high level of inattention both prior to and after school entry, whereas, for girls with dyslexia, inattention increased after school entry. Girls with dyslexia reached the levels of boys with dyslexia by the age of 9 years. Inattention was not associated with family risk; however, the family risk children without dyslexia did not have elevated levels of inattention. Similarly, more task-avoidant behavior was reported by parents and teachers of children with poor reading skills in early grades [102] and poorer pre-reading skills [103]. However, the associations between task avoidance and skills did not remain in the participants’ self-reports at later ages [102]. Similarly to attention, task avoidance appeared to be associated with reading skills rather than with family risk. In addition, task avoidance was associated with the temperament features of high effortful control and high negative affectivity [103]. Interestingly, the findings of Eklund et al. [80] further suggested that low levels of task avoidance may act as a protective factor: the JLD children who did not have dyslexia in Grade 2 despite difficulties in early cognitive development had no difficulties focusing on tasks. On the contrary, the children who had dyslexia despite typical cognitive development were reported by their parents and teachers to have difficulties focusing on tasks.

#### 3.2.5. Reading Development—From Decoding and Fluent Reading to Comprehension

One of the strengths of the JLD project is that the participants have been followed over many years. This has increased our knowledge about the long-term development of reading. Our findings have suggested that reading fluency develops until adolescence, that reading difficulties are stable for many but not for all, that reading fluency and reading comprehension associations change across time, and that the predictors of reading skills are also different at different ages.

Despite the rapid change of skills during development, correlations across time were high, particularly for reading speed. Stability in the rank positions of individuals was shown to be high from Grade 2 to Grade 8 in reading speed (Pearson *r* = 0.78), whereas only moderate associations were found in reading and spelling accuracy, partly because these measures approached ceiling levels [29]. In other words, even the individuals with dyslexia in Grade 2 reached, on average, a high accuracy level (>90%) by Grade 3, when the items to be read were words. In pseudoword reading, as well as in spelling, they continued to struggle even in Grade 8. Our findings thus suggest that in a regular orthography most children with dyslexia will eventually acquire sufficiently good skills in phonemic awareness to enable an accurate, albeit rather slow, decoding ability. Consequently, the individuals with dyslexia in Grade 2 showed a constant lag in reading speed compared to children without dyslexia in Grade 2. In Grade 8, the reading speed of these children was at the level of typical readers in Grade 3 (with no dyslexia in Grade 2), thus lagging by about five years in development. Importantly, however, the growth rate was practically equal to that of typical readers, and no evidence was found for lagging further behind. We found pieces of evidence for several possible explanations for the lower reading speed of children with dyslexia in Grade 2. Comparisons of reading speed in different tasks (word list, pseudoword list, and text reading) suggested that the children seemed to rely longer on sub-lexical reading processes, to have difficulties in the use of their orthographic lexicon, and therefore to shift later to using lexical reading processes compared to typical readers in Grade 2. In addition, these children seemed to be less capable of using contextual cues in text reading.

Despite the high correlations between Grade 2 and Grade 8 reading fluency, when looking at the stability of diagnosis at the individual level, we found moderate instability of dyslexia status across these time points, which cannot be fully due to random changes across cut-offs [30]. We separated three sub-groups of children based on their dyslexia status into one or two evaluation time points and their different developmental trajectories. The Persistent group met the criteria for dyslexia based on reading fluency both in Grade 2 and Grade 8 and comprised the majority (40%) of these children. This group of children showed not only persistent reading difficulties but also long-lasting deficiencies in preschool-age cognitive foundations of reading (RAN, letter knowledge, verbal short-term memory), but not in early phonological awareness or vocabulary. The Late-Emerging subgroup (33%, mainly boys) showed no dyslexia in Grade 2 but fulfilled the criteria for dyslexia in Grade 8. Strikingly, this group showed especially poor performance in preschool-age rapid naming, the core predictor of reading speed, e.g., [24]. Additionally, their parents had the most severe difficulties in rapid naming, a finding that suggests a strong genetic liability. The third subgroup, the Resolving (27%, mainly girls), included children with dyslexia in Grade 2 but not in Grade 8. They showed widespread deficiencies in preschool-age cognitive skills, but they disappeared after entering school. This suggests that school entry resulted in a clear improvement in their environmental support related to reading skill development. Another possibility is that they suffered from a developmental delay rather than a permanent cognitive deficit. The different developmental trajectories of these three groups highlight the importance of continuous follow-up and support of reading skills up to adolescence.

Finally, we have also examined the development of reading comprehension as part of the JLD. Although reading comprehension difficulties are not part of the dyslexia criteria, reading comprehension is important for learning and functioning in modern societies. We were also interested to see whether familial risk of dyslexia and children’s dyslexia play a major role also in the development of reading comprehension difficulties. Furthermore, the longitudinal design allowed us to examine early skills that may predict the development of reading comprehension. In a study on early reading fluency and comprehension development in Grades 1–2 [89], five subgroups with dissimilar developmental profiles in reading fluency and reading comprehension were identified. The majority of the children had good (10.6%) or average (41.5%) skills in both word recognition and reading comprehension, while three groups were characterized with differently deficient reading skills. Slow decoders (24.6%) reached the level of their classmates in reading comprehension by the end of Grade 2, although they were somewhat below the average in terms of word recognition fluency. Poor comprehenders (10.7%) showed an increasing lag over time in reading comprehension despite their age-equivalent word recognition fluency skills. Poor readers (12.6%) showed poor performance both in word recognition fluency and in reading comprehension throughout Grades 1 and 2. Children with family risk for dyslexia were overrepresented in Slow decoders, and in Poor readers there were twice as many children with family risk as children without family risk (17.3% vs. 9.2%). Slow decoders and Poor readers showed widespread deficiencies in early reading-related cognitive skills (rapid naming, phonological awareness, and letter knowledge). In addition, Poor readers’ language skills at the preschool age were below average, which was the striking characteristic of Poor comprehenders. A similar dissociation of reading fluency and reading comprehension was found in adolescence [95], and similar results have been reported in other samples across orthographies, e.g., [104,105,106]. Overall, our data verified the existence of the differential reading subtypes [107,108,109]. Thus, we found support for the partly differential origin of word recognition and reading comprehension and the central role of language skills in the development of the latter.

The crucial role of language skills in reading comprehension was further validated by structural equation modelling related to the prediction of PISA reading at the age of 15 years [110]. Language skills, including expressive and receptive vocabulary as well as morphology, measured at 2.5–5.5 years directly explained a great deal of the variance in PISA reading (53% and 31%, with and without family risk, respectively), while pre-literacy skills (phonological awareness, rapid naming, and letter knowledge) indirectly explained it through reading fluency to a lesser extent (15% and 13%, with and without family risk, respectively). Children with family risk had compromised skills in both reading fluency and reading comprehension. However, when compared to children without family risk, the effect sizes for reading fluency were moderate to large but small for reading comprehension. The importance of language skills for reading comprehension was also highlighted in our study, where the effect of early language delay on later reading skills was examined [111]. Only at-risk children with a delay in both receptive and expressive language at 2–2.5 years showed poor performance in PISA reading at the age of 15 years. The poor reading comprehension skills of this group were already visible in Grade 2. Early delay in expressive language alone did not result in impaired PISA performance, nor did it affect reading fluency in Grades 2, 3, or 8. Taken together, family risk alone resulted in compromised reading fluency, but when the risk was accompanied by early receptive and expressive language delay, reading comprehension and fluency were both compromised.

#### 3.2.6. Prevention and Intervention

Inefficient processing of speech sounds, as outlined above, is common for Finnish children at risk of familial dyslexia and hinders the acquisition of the accurate and efficient decoding skills needed for discriminating acoustically similar letter sounds, e.g., [14,15,16,17,18,63,78]. Inspired by and based on these results, GraphoGame, an enjoyable serious digital learning game, has been developed to support the acquisition of decoding by training the letter–sound correspondences and basic reading skills, e.g., [112,113,114,115,116,117]. A recent meta-analysis on GraphoGame studies revealed that playing the game alone is not effective enough. However, with high adult interaction, training effects with a medium effect size can be achieved, which suggests that GraphoGame would be useful in a classroom context [118]. There is also evidence that GraphoGame could be effective in non-transparent languages, such as English, when the spoken and written units to be connected are larger [119]. In addition, the game has shown promise for helping second language readers [120]. Today, GraphoGame is in use by more than 1.5 million children all over the world (please see Lyytinen et al. [121] for the most recent summary of global research and use).

## 4. Lessons Learned in JLD—Combining Brain, Behavioral, Environmental, and Intervention-Related Findings to Solve the Mysteries

**Figure 4:**
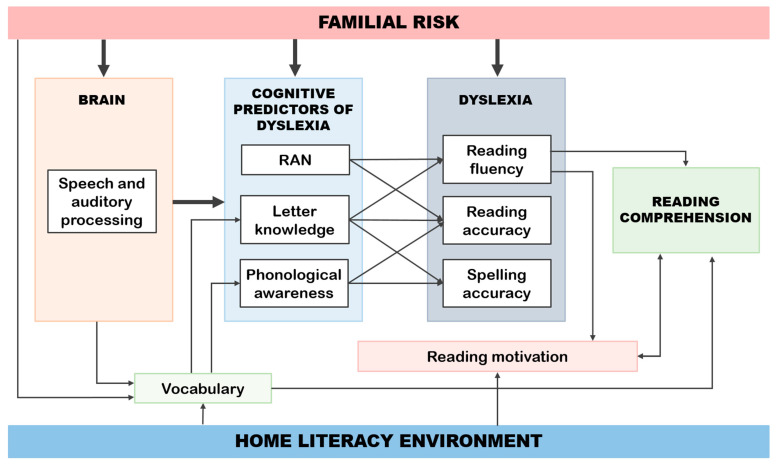
The predictive connections found between the key measures in the Jyväskylä Longitudinal Study of Dyslexia (JLD) project. The thick arrows denote connections to all individual skills or measures mentioned under the domain.

In this section and in Figure 4, we summarize the main findings and key conclusions of the JLD project. Our findings agree with the idea of neuroconstructivism that basic-level deficits may have cascading effects that alter the interactions within and between networks and may constrain the emergence of functions in other domains [122]. This means that brain-level precursors (e.g., auditory insensitivity, formation of phonological representations) may affect later developing functions (e.g., rapid naming, phonological awareness, letter knowledge), which will be predictors of reading struggles at the beginning of school. These difficulties in reading accuracy and fluency together with deficiencies in language skills modified by environmental factors may later expose the child to difficulties in reading comprehension as well. Based on the JLD studies, we suggest that our developmental and multi-level (genetic, neural, cognitive, behavioral, and environmental) approach using a prospective familial risk design is an excellent yet challenging way to solve the mysteries of dyslexia. The results of the study have also advanced the development of preventive training tools, which may support children to overcome the deficits leading to reading problems at any level.

### 4.1. Infant Brain Responses Are Associated with Later Reading Skills

The brain responses of infants at risk of dyslexia and those of the control children already differ in the first week of life and remain persistently different later despite environmental factors affecting their speech perception [14,15,16,18,19,20,64]. These hemispheric and amplitude differences between groups in brain responses indicate early deficits in auditory and speech sound processing in at-risk infants, especially those who later became dyslexics. Overall, these findings suggest that speech perception deficits precede reading acquisition, learning of letter–speech sound associations, and complex phonological skill development. The findings also indicate that, along with other risk factors, early brain responses could be helpful for the early identification of children at risk of poor reading success or dyslexia [15,22,36,61,62,63]. In JLD, we have shown, for example, that brain activation measured at six months to the pseudoword /ata/ predicted over 40% of the reading speed score at adolescence, and it even improved the prediction of reading speed beyond the preschool measures of phonology, letter naming, and verbal short-term memory [63]. In addition, we have shown that newborn ERPs improve the prediction of rapid naming, phonological processing, and letter naming skills at preschool age on top of family risk status [62]. Similar findings were reported in a Swiss study, in which ERP measures in kindergarten increased the Grade 2 and Grade 3 reading score prediction from 15–16% to 32–36% [65]. 

The use of brain measures as predictors for identifying children who might later benefit from interventions is one practical application of brain research on dyslexia. However, several factors need to be considered before this would be feasible. Currently, it is challenging to use brain measures as predictors because of the relatively low signal-to-noise ratio of brain responses at individual level, which makes the standardization of neural indicators difficult; thus, the results are primarily interpretable at the group level. Brain measures are also relatively expensive to obtain, and the difficulty in obtaining sufficiently large samples to define cut-off points for standardization has held back this line of research. The sample sizes in all ERP studies with family risk groups are relatively small (in our infant ERP studies, generally ranging from 20 to 40 per group) and therefore vulnerable to random variation and a lack of statistical power. Further, brain research has identified a diverse set of brain indices associated with dyslexia-related cognitive measures and dyslexia, such as the topographic distribution of ERP responses, amplitude of obligatory ERPs, and change detection-related ERPs [58,59,63]. Designing robust stimulation paradigms and using new analysis approaches that can extract specific brain responses could improve this situation. Paradigm development could include, for example, an examination of stimulation rate effects on brain responses, optimal stimulus contrasts, and task selection for the participant (e.g., passive perception or active comparison task). These could be used to boost the signal of interest and optimize the discrimination power of the brain measures. Currently, the research has focused on examining the predictors at the group level, e.g., [22,62,63,65].

Consequently, in the future, broader international co-operational research projects with multisite data collection are needed to confirm the findings. It would also be worthwhile to consider whether it would be possible to find common indices to combine some or all of the early ERP datasets to get larger samples. This would enable, for example, estimation of how many at-risk infants and children show speech perception differences in brain measures, how language specific the findings are, or whether there are common general underlying mechanisms explaining the results. This also poses a challenge for future studies: before brain measures can be used for identification, reliable individual brain measures must be developed. The usability of ERPs for early identification still requires basic research and validation in order to have good estimates of how much the early identification of reading problems could be improved by the use of brain measures. 

### 4.2. There Are Multiple Cognitive and Oral Language Predictors of Reading Skills

Our findings suggest that there are four key cognitive skills that predict reading development and dyslexia years before school entry: oral language skills, phonological awareness, letter knowledge, and rapid automatized naming. These same skills also differentiated children with and without family risk from 3.5 years onwards (i.e., from the time point assessment of the aforementioned skills began [70]). Phonological awareness, letter knowledge, and rapid automatized naming turned out to be the key cognitive predictors of dyslexia at the early stage of school [24]. Notably, however, family risk improved the accuracy of identifying children with dyslexia over and above these cognitive key predictors. With those three skills and familial risk we were able to identify children with dyslexia reliably starting from the age of 3.5 years. Individual risk for dyslexia was found to be a combination of phonological awareness, RAN, letter knowledge, and familial risk, but it was not the same for all children. In line with this, subgroups with different developmental trajectories in cognitive skills prior to school entry leading to dyslexia were found [11]. For example, the children in the Dysfluent group were characterized by slow naming speed and children in the Unexpected group by poor letter knowledge, while children in the Declining group showed a wide array of deficient cognitive skills. The effect of deficient phonological awareness, however, was restricted to the very early phase of reading acquisition, after which it mainly affected spelling, but only for children with family risk [77]. It should be noted that receptive and expressive language skills also differentiated the children with and without dyslexia from age 2 onward [70]. However, oral language skills did not make a unique contribution to the prediction of dyslexia after considering the effects of the three key cognitive predictors [24]. Receptive vocabulary had a strong effect on the development of reading comprehension until adolescence [89,110,111,123].

These findings suggest that, although it is possible to pinpoint the key cognitive deficits that predict subsequent reading development and dyslexia, the cognitive profiles are not the same for each individual, which supports the multiple deficit view on dyslexia [7,8]. This is well fitted with the fact that reading skills and difficulties do not manifest similarly across individuals, as shown in many other samples as well, e.g., [104,105,106]. For example, some experience difficulties in grasping the principles of decoding, others struggle in the development of automatization of decoding, while for others reading comprehension is the key difficulty. This is not surprising, of course, as reading is a complex skill that requires multiple cognitive processes and is impacted by multiple factors during the process and over development.

### 4.3. Reading Difficulty Profiles Vary with Age and between Individuals

Our findings suggest that reading speed develops until adolescence, although reading accuracy hits a ceiling very early. Due to the combination of the transparent Finnish orthography and efficient reading teaching in schools, most of the children learn to read during the first semester, and even the majority of children with dyslexia will eventually acquire an accurate decoding ability. Inaccuracy is typically only seen when spelling or when reading unfamiliar or rare words with particularly difficult structures. Therefore, reading difficulties in Finnish mainly manifest in reading fluency. In JLD, children with dyslexia showed a constant lag in reading speed but developed at a similar speed as their peers. However, when we explored the stability of the dyslexia status of each individual, the developmental differences were identified. We found three groups with different developmental trajectories (persistent, late-emerging, and resolving) in reading fluency between Grade 2 and Grade 8, which resulted in moderate instability of dyslexia status. The groups also had expectedly differential cognitive profiles, which partially explained the differential trajectories. These findings highlight the importance of continuous follow-ups and support of reading skills until adolescence. Moreover, we should not stop following reading skill development after the early school years, as an early dyslexia diagnosis may not hold over many years, and importantly, there are also late-emerging cases that may not be spotted early on. While the late-emerging cases are able to learn decoding rules and perform close to average during the early school years in reading tests, they are not able to develop automaticity in reading. Such a developmental profile can be problematic in learning and educational paths, particularly if the difficulties are not identified. 

Furthermore, learning to read does not end when a child has learned to decode rapidly and accurately, as the final goal of learning to read is reading comprehension. According to our findings, early reading comprehension is naturally dependent on reading fluency skills. The importance of reading fluency in reading comprehension started to decline, however, from Grade 2 onwards. Similar findings have been reported in many studies, but this differentiation of reading skills seems to happen somewhat earlier in transparent orthographies, such as Finnish. This is likely due to the quick process of reading acquisition. Once children become fluent enough in reading, they can use their cognitive resources for reading comprehension rather than focusing on decoding letters to sounds, e.g., [104,108]. Reflecting on this separation, the familial risk of dyslexia was a strong risk factor for reading comprehension difficulties only during the very early phases of reading acquisition. The key early cognitive risk factor for reading comprehension difficulties was also different from the predictors of dyslexia, early oral language, and receptive vocabulary in particular. Children with difficulties in oral language comprehension as early as 2.5 years were clearly at higher risk than their peers of developing reading comprehension difficulties.

Overall, it thus seems that there were four main pipelines for reading difficulties in this sample, three for dyslexia, and one for reading comprehension. First, difficulties in phonological processing often resulted in reading and spelling accuracy difficulties. However, the difficulties in reading accuracy were short-lived in a transparent orthography, and phonological processing skills had a smaller effect on reading skills than in many other orthographies. Second, slow performance in rapid automatized naming (possibly indicating slow processing of linguistic material) often resulted in automatization difficulties and thus a very slow reading speed. Third, letter knowledge predicted reading accuracy and speed. As letter knowledge is very close to reading in a transparent orthography, its key role as a predictor is expected. Fourth, comprehension of oral language often resulted in reading comprehension difficulties. This is also expected because, after the letter–phoneme conversion rules are fully automatized, reading comprehension should be a cognitive process close to language comprehension.

### 4.4. The Home Environment Is Associated with the Development of Oral Language, Letter Knowledge, and Reading Motivation

The home environment was examined as a potential mediator or moderator of familial risk of dyslexia. Had it been different in the study groups, it would have meant that familial risk was affecting children’s development through a less supportive home environment. However, in JLD, the home environment was found to be similar in the groups with and without familial risk for dyslexia. Although parents’ own interest in reading was less positive in the family risk group, they read as much with their children, went to the library as often, had as many books at home, and taught literacy skills to their children as often as the control group parents. However, it is possible that the home environment acted as a moderator or protective factor. Of the various home environment factors, the ones that included active interaction with children were found to be associated with children’s skills: parent–child shared reading was associated with oral language development, e.g., [69], and further with the child’s reading comprehension skills at a later age [90,91]. Parental teaching of letters and reading predicted the development of letter knowledge [68]. The HLE factors not involving interaction between parents and children (e.g., library visits, number of books at home, or parent’s own reading interest) were not related with children’s skill development. 

As the correlations between shared reading and vocabulary were found to be higher among the family risk than among the control children [69], it seems that reading with at-risk children may be particularly beneficial. Interestingly, this may suggest that shared reading can even act as a protective factor supporting oral language development and through oral language support later reading comprehension as well. It should also be noted that parent–child shared reading was found to support children’s reading interest. Therefore, as reading interest was in turn found to support reading development, shared reading potentially, via this indirect route, also supports reading. It is important to acknowledge, however, that the interactions in homes are not unidirectional from parents to children. Children choose activities, and their characteristics evoke reactions from the environment. Therefore, correlations between children’s characteristics and the home environment, even longitudinal ones, do not fully prove the impact of home activities on children’s skills. Although we did not identify significant effects from children’s interest or skill measures to home environment factors, we do acknowledge the potential role of the gene–environment interaction and correlation mechanism in strengthening the correlations, e.g., [9].

### 4.5. Reading Motivation Is Important in the Development of Reading Fluency and Comprehension

With regard to reading motivation, it seems that the JLD parents with reading difficulties did not directly pass their own lower reading interest to their children, as the children with and without family risk were not found to differ in reading interest at any time point, from toddlers to adolescents [68,69,88,89,91]. However, reading interest was found to be associated with reading skills in both study groups. At school age, children’s reading interest was found to be reciprocally associated with reading fluency and reading comprehension [89,91]. During the early grades, poor reading skills seemed to constrain children’s possibilities to enjoy reading, while during the later school years reading more leads to better reading comprehension skills. In adolescence, reading comprehension was associated with various problems in school motivation [100]. As reading more did not seem to support reading fluency development, the link appears to operate via other routes, most likely oral language. However, the low levels of task avoidance may act as a promotive factor for reading fluency: among the children who had early cognitive difficulties, dyslexia was less often identified if the children had low levels of task avoidance [80].

The role of reading motivation may be particularly important for the children at high risk of reading difficulties. This is because individuals with reading difficulties will need to invest considerably more time and effort than their peers; first to acquire reading skills and later to be able to learn by reading. Those who choose to engage often in reading activities have frequent possibilities to learn various reading-related skills as well as to gain knowledge through reading. As reading motivation is associated with reading comprehension in particular, it is possible that, over the years, reading engagement acts as a compensation mechanism. It may be speculated then that the impact of dyslexia on education could become diluted for those who have high reading motivation (i.e., enjoy reading, engage in reading often, and do not have high levels of inattention or task avoidance when facing difficult tasks) despite early reading difficulties. To secure learning opportunities for all, it is of utmost importance to support children’s reading interest, particularly for those who have reading difficulties.

### 4.6. New Intervention Methods for Preventing and Solving Reading Difficulties

The final aim of the JLD project was to use the gained knowledge to develop an intervention. The development has been based on the findings that auditory insensitivity to acoustically similar letter sounds and the phonemic length-related aspects in reading and spelling are the most difficult for Finnish children and hinder the acquisition of accurate decoding skills [17,114]. Based on these findings, an enjoyable serious digital game called GraphoGame has been developed, and it has already been translated into tens of languages to train basic reading skills. In GraphoGame technology, the association learning principles have been applied to help children learn to connect the units of spoken language to equivalent written language units (for an extensive explanation, see [112]). According to a meta-analysis of intervention approaches for children and adolescents with reading disabilities, the treatments that teach letter–sound correspondences and decoding strategies were the only interventions with statistically significant efficacy in terms of improving reading and spelling performance [124]. 

We have also started to examine brain processes during the learning of audio-visual associations, with the ultimate goal to understand why some individuals learn the associations faster than others [125,126]. This has begun by examining the feasibility of observing changes in brain measures during training sessions. The training has focused on learning new character–speech sound associations. Interestingly, changes could be observed in both EEG- and MEG-based brain measures during 40-min training sessions. For example, when adult participants were asked to learn associations between unfamiliar symbols and heard syllables by trial and error, changes in brain responses obtained with MEG originating from the temporo-occipital and frontal cortices were observed after 10 min of training [125]. In a comparable experiment where Georgian characters and Finnish speech sound associations were taught to the participants, we again observed rapid changes in brain activity measured with MEG during the learning situation in the posterior superior temporal sulcus [126]. These observed changes could be considered as parallel markers of learning, along with changes in reaction times and accuracy scores. Further, we observed a reversed effect on brain response strength of learning cues given during the training compared to the responses to the audio-visual pairs. The cues gave information about whether the presented character–phoneme pair was the correct or incorrect pairing. The brain response contrast to the cues was largest at the beginning of training and reduced after the correct associations were learned, likely indicating attention allocation to elements crucial for the task. These studies show that brain measures could be used to study the processes that are used during different intervention programs. If these measures turn out to be predictive of longer-term learning outcomes, they could be used as indicators of important cognitive processes that can be fine-tuned in the training programs. The next steps in utilizing brain research in these kinds of applications would be to examine the online training processes in children and to conduct follow-up studies on how well the brain measures are associated with later success in reading acquisition.

## 5. Conclusions

Among other research, the JLD project has revealed that it is possible to identify children at risk of severe reading problems early on. This means that support for reading development can be given in a preventive way; that is, by starting support even before the beginning of formal reading instruction. Early support is important, as it seems to be more efficient than late-onset support. In addition, we should try to avoid the accumulation of failure experiences for children, which could lead to lower learning motivation and well-being, thereby creating negative feedback loops or so-called vicious circles. Especially in children who have cognitive deficits and are therefore in need of a lot of practice, reduced motivation may lead to less exposure to written material and thus training, ultimately resulting in an inability to achieve an adequate level of learning and their full potential as learners. Our long-term follow-up has shown that not all reading difficulties emerge in the reading acquisition phase. The purpose of reading is to mediate the meaning from the written material. Some children who overcome the problems associated with the first step of learning to decode may later face difficulties in reaching sufficient fluency or in comprehending the content. For some children, reading comprehension may require special support. Hence, we cannot identify all children with reading difficulties prior to school entry or even after a few years in school. However, continued follow-up of development is advisable. Finally, as individuals differ in their skill profiles and developmental trajectories, the support needs to be individually planned to match the child’s skills, interests, and developmental phase.

## Data Availability

No new data were created or analyzed in this study. Data sharing is not applicable to this article.

## Supplementary Materials

The following are available online at https://www.mdpi.com/article/10.3390/brainsci11040427/s1: Table S1: List of assessments at each measurement point of the JLD project, Table S2: Summary of the technical details in EEG measurements.
