# Supplementary material for: Unveiling the Mysteries of Dyslexia—Lessons Learned from the Prospective Jyväskylä Longitudinal Study of Dyslexia
Source: Brain Sci. 2021 Mar 27;11(4):427. doi: 10.3390/brainsci11040427 (PMC8066413; doi:10.3390/brainsci11040427)
Supplement: Supplementary file 1 [file brainsci-11-00427-s001.zip › Table S1_List of assessments at each measurement point of the JLD project .pdf]

Jyväskylä Longitudinal Study of Dyslexia, JLD: Measures

Age	Content/measure	Data
1-5 days<br>Hospital	Brain responses to speech/nonspeech stimuli	ERP + Heart rate
**Age**	**Content/measure**	**Data**
6 months	Brain responses to speech/nonspeech stimuli	ERP + Heart rate
	Categorical perception of speech stimuli	Head turn
	Parent-child interaction & motor imitation	Videotape
	Visual recognition memory	Test
**Age**	**Content/measure**	**Data**
0 –12 months	Developmental milestones in<br>1) vocalization,<br>2) speech comprehension,<br>3) motor & fine motor development	Parent diary: checklists
1, 6 & 12 mo	Infant Behavior Questionnaire: Temperament	Parental report
4 –24 months	Vocalization and speech in interactional situations at home (subsample, n = 60; monthly videotapings)	Video-tapings by parents at home
**Age**	**Content/measure**	**Data**
12 months	MacArthur Communicative Development Inventory (MCDI*):*<br>Vocabulary comprehension and production	Parental report
**Age**	**Content/measure**	**Data**
14 months	Interaction: Parent-child free play<br>Joint book reading	Videotape
Lab	Structured play	Videotape + DAT recording
	Observation of wariness in a novel situation: Temperament	Videotape
	MacArthur Communicative Development Inventory	Parental report
**Age**	**Content/measure**	**Data**
18 months	Reynell Developmental Language Scales*:* Verbal comprehension & expressive language	Test
Lab	Structured play	Videotape
	Word-imitation task	Videotape + DAT recording
	MacArthur Communicative Development Inventory (MCDI*):*<br>Vocabulary production, use of suffixes, Maximum Sentence Length	Parental report
**Age**	**Content/measure**	**Data**
24 months	Bayley Scales of Infant Development II*:* MDI, PDI, Language composite, Expressive language, Receptive language	Videotape + DAT recording
Lab	Interaction: Parent-child free play<br>Joint book reading	Videotape + DAT recording
	MacArthur Communicative Development Inventory	Parental report
	Questionnaire: Shared reading and joint activities	Parental report

Age	Content/measure	Data
2.5 years	Reynell Developmental Language Scales	Test
Home	Picture naming task: phonological accuracy of speech production, rhyme repetition	Videotape + audiotape
	Comprehension of inflectional forms	Test
	Speech elicited in structured play	Videotape
	MacA*r*thur Communicative Development Inventory	Parental report

Age	Content/measure	Data
3 years	Bayley Scales of Infant Development II<br>Controls: n = 60; At risk: n = 33 (severe dyslexia)	Test
Subgroup	Boston Naming Test	Test
Lab	Parent-child problem solving	Videotape
	Parent-child book reading	Videotape

Age	Content/measure	Data
3.5 years	*Language clubs*. October 1996 ∃ May 1999. 10 groups, n = 66<br>Group activity for children and parents (involving individual child assessments). Groups of 6-8 children met once a week for 7 to 9 times.	Videotape

Age	Content/measure	Data
3.5 years<br>Lab<br>2 visits	<u>Phonological awareness</u><br>1) Word-level segment identification<br>2) Syllable-level segment identification<br>3) Synthesis of phonological units<br>4) Continuation of phonological units	Computer-aided assessment with touch screen (AHeps-Kups Land@)
	<u>Phonological processing</u><br>1) Perception of time-compressed words<br>2) RAN: Rapid naming of individually presented pictures<br>3) Repetition of pseudowords	Computer-aided assessment with touch screen (AHeps-Kups Land@)
	NEPSY - *A Developmental Neuropsychological Assessment*<br>Korkman, M., Kirk, U., & Kemp, S. (1998)<br><br>*Attention/Executive*: Visual attention. *Language*: Naming of body parts; Phonological processing A; Comprehension of instructions; Repetition of nonsense words. *Sensorimotor*: Visuomotor precision; Finger discrimination. *Visuospatial)*: Design copying; Block construction; Picture recognition. *Memory*: Immediate memory for faces; Narrative memory; Sentence repetition.	Test
	<u>Orthographic skills</u><br>1) Identification of letters<br>2) Naming letters<br>3) Logographic identification of words<br>4) Visual matching<br>5) Identification and writing of one=s own name	Test
	<u>Other language measures</u>:<br>1) Boston Naming Test<br>2) Peabody Picture Vocabulary Test (adaptively)<br>3) Morphology Test<br>4) Digit Span<br>5) Rapid naming (RAN): Objects	Test
	Parent-child book reading	Videotape
	Movement-ABC Test<br>Automatisation: dual task (balance & naming)	Test + videotape

Age	Content/measure	Data
4 years<br><br>Parent report	<u>Parent questionnaires</u><br>1) Shared reading & joint activities with the child<br>2) Day care & peer relations<br>3) Parenting attitudes, mood, stress, employment<br>4) *Behavior Assessment System for Children (BASC)* Reynolds, C. R., & Kamphaus, R. W. (1992). Adaptability, Social skills, Attention problems, Hyperactivity, Aggression, Anxiety, Depression, Somatization, Atypicality, Withdrawal<br><br>5) Child Behavior Questionnaire: Temperament	 Parental report

Age	Content/measure	Data
4.5 years<br><br>Lab	<u>Phonological awareness</u><br>1) Syllable/phoneme-level segment identification(16 items)<br>2) Synthesis of phonological units (12 items; e.g., *m-u-n-a*)<br>3) Continuation of phonological units (12 items; e.g., *ki-*)<br>4) Say just a little bit (8 items; e.g., *puhelin*)<br>5) Odd one out: Rhyme (8 items; e.g., *raha-kynä-saha*)<br>6) Odd one out: Alliteration (8 items: e.g., *peikko-peitto-nappi*)	 Computer-aided assessment with touch screen (AHeps-Kups Land@)
	<u>Phonological processing</u><br>1) Perception of time-compressed words (18 items)<br>2) Rapid naming of individually presented pictures<br>3) Repetition of pseudowords (18; e.g., *paohki, jomettö*)<br>4) Perception of non-speech sounds & sound patterns	 Computer-aided assessment with touch screen (AHeps-Kups Land@)
	<u>Other phonological awareness tasks</u>:<br>1) Initial phoneme recognition (9 items; *R ∃ tuoli, raha, keinu, juna*)<br>2) Initial phoneme production (8 items; e.g., *siili*)<br>3) Rhyme production (10*; Kissa istui puussa, iso kala* C *)*<br>4) Word segmentation (14 items; e.g., *pain-sa, s-u-o*)	 Test
	Morphology Test	Test
	<u>Orthographic skills</u><br>1) Identification of letters (8 items)<br>2) Logographic identification of words (6 items; e.g., *Muumi*)<br>3) Visual matching (10 items; e.g., *RU, π, nas*)<br>4) Identification of one=s own name	 Test
	<u>Reading & Spelling</u><br>1) Naming letters (23 letters)<br>2) Reading words/nonwords (4 + 4 items; *isä, suu, talo, rui / pallo, kaunis, siellä, olus*)<br>3) Spelling words/nonwords (5 + 4 items: *äiti, auto, tie, nalle, usa / koira, sakset, temppu, ryhe*)<br>4) Writing one’s own name	 Test
	Frog Story (child=s narrative based on a series of pictures)	Test & videotape
	Print exposure (e.g., letters & phonemes)	Parental report

Age	Content/measure	Data
5 years	**Peabody Picture Vocabulary Test**	**Test**
	**Morphology Test**	**Test**
	**<u>Memory</u>**	
	**1) Digit Span,**	**Computer test**
	**2) Span for syllables**	**Computer test**
	**3) Memory for spatial sequences: Corsi blocks**	**Test**
	**<u>Accuracy of representations</u>:**<br>**Perception of phonological variants of words and non-words**	**Computer test**
	**<u>Orthographic skills</u>**	
	**1) Identification of letters (8 items)**	**Test**
	**2) Logographic identification of words (6 items; e.g., *Muumi*)**	
	**3) Visual matching (10 items; e.g., *RU, π, nas*)**	
	**4) Identification of one=s own name**	
	**<u>Reading & spelling</u>**	
	**1) Naming letters (23 letters)**	**Test**
	**2) Reading words/nonwords (4 + 4 items; *isä, suu, talo, rui* / *pallo, kaunis, siellä, olus*)**	
	**3) Spelling words/nonwords (5 + 4 items: *äiti, auto, tie, nalle, usa / koira, sakset, temppu, ryhe*)**	
	**WPPSI-R (Wechsler Preschool and Primary Scale of Intelligence-R; Wechsler, 1989): 3 + 3 short form**	**Test**
	**1) Vocabulary**	
	**2) Arithmetic**	
	**3) Comprehension**	
	**4) Block Design**	
	**5) Object Assembly**	
	**6) Picture Completion**	
	**<u>Parent-child interaction</u>: Constructing a story sequence using series of pictures with temporal order**	**Videotape**
	**<u>Questionnaires</u>:**	
	**1) Shared reading & joint activities with the child**	**Parental report**
	**2) Day care**	
	**3) Behavior Assessment System for Children (BASC)**	

Age	Content/measure	Data
5.5 years (2 visits)	NEPSY – Developmental Neuropsychological Assessment<br>Korkman, M., Kirk, U., & Kemp, S. (1998)<br><br>*Attention/Executive:* Visual attention; Tower; Auditory attention; Knock and tap. *Language:* Phonological processing; Comprehension of instructions; Repetition of nonsense words; Oromotor sequences; Verbal fluency. *Visuospatial:* Design copying. *Sensorimotor:* Visuomotor precision. *Memory:* Narrative memory; Sentence repetition; Memory for names; Immediate & delayed memory for faces.	Test
	Boston Naming Test	Test
	<u>Phonological awareness</u><br>1) Initial phoneme recognition<br>2) Initial phoneme production<br>3) Rhyme production<br>4) Word segmentation	Test
	5) Syllable/phoneme-level segment identification	Computer test
	6) Synthesis of phonological units	Computer test
	<u>Association learning</u><br>1) Audio-visual association<br>2) Audio-phonemic association<br>3) Visual-visual association<br>4) Memory association	Computer test
	RAN: Objects & colors	Test
	<u>Orthographic skills</u><br>1) Identification of letters<br>2) Logographic identification of words<br>3) Visual matching	Test
	<u>Reading & Spelling</u><br>1) Naming letters<br>2) Letter to phoneme production<br>3) Reading words/nonwords<br>4) Spelling words/nonwords<br>5) Synthesis and segmentation of nonsense syllables<br>6) Identification of word meaning	Test

Age	Content/measure	
6 years	<u>Parent questionnaires</u><br>1) Shared reading & joint activities with the child<br>2) Day care<br>3) Social skills, social network<br>4) Behavior Assessment System for Children (BASC)<br>5) Parenting attitudes, mood, strategies, employment	Parental report
	<u>Preschool teacher questionnaire</u><br>1) Motivation<br>2) Achievement<br>3) Strategies	Preschool teacher ratings

Age	Content/measure	Data
6.5 years<br>(2 visits)	<u>Phonological awareness</u>	
	1) Initial phoneme recognition	Test
	2) Initial phoneme production and deletion (Poskiparta ym.)	
	3) Syllable deletion (Poskiparta ym.)	
	4) Word segmentation	
	5) Syllable/phoneme-level segment identification	Computer test
	6) Continuation of phonological unit	Computer test
	Non-word repetition	Computer test
	<u>Association learning</u>	
	1) Audio-visual association	Computer test
	2) Audio-phonemic association	
	3) Visual-visual association	
	4) Memory association	
	Digit span	Test
	Rapid naming (RAN): Objects, colors, numbers, letters	Test
	<u>Reading & Spelling</u>	
	1) Naming letters	Test
	2) Reading words/nonwords	
	3) Spelling words/nonwords	
	4) Synthesis and segmentation of nonsense syllables	
	5) Identification of word meaning	
	6) Text reading	
	Movement ABC Test	Test
	Child interview: Strategies & attributions	Self-evaluation
	Ratings: Child=s strategies	Rating by the tester
	Auditory discrimination: ERP - speech & non-speech	Test
	Auditory behavioral categorisation - speech & non-speech	Test

Age	Content/measure	Data
6.5 years ∃<br><br>Case studies	<u>Interventions</u><br>1) Phonological skills<br>2) Naming<br>3) Family School – group intervention for children with overactivity and their parents.<br>Group 1, Spring 2001: 5 children + 5 parents,<br>Group 2, Fall 2001: 4 children + 6 parents	Tests, videotape

Age	Content/measure	Data
7.0 years	<u>Questionnaires</u>	

GRADE 1: August, Individual assessment	Data
**Phonological awareness**	
1) Phoneme blending (Poskiparta ym.; *p-u-u*)	Tests
2) Syllable deletion (Poskiparta ym.; *paluu* ∃ *luu*)	
3) Initial phoneme production & deletion (Poskiparta ym.; *asuu* ∃ *suu*)	
4) Phonological Same Different Matching Task: Syllable, rime & phoneme level (*poika - sulka; varvas – karhu; hattu - helmi*)	Cognitive Workshop
5) Phonological Common Unit Task: Syllable, rime & phoneme level (*lehmä – lehti; leija - neiti; noita - kukka*)	Cognitive Workshop
**Reading**	
1) Letter naming (29 letters)	Cognitive Workshop
2) Content words (9 words)	
3) Function words (9 words)	
4) Syllables (9; e.g, *eh, vis, li, nä*)	
5) Bi-syllabic nonwords (9; e.g., *värö, ame, hopa*)	
6) Complex nonwords (9; e.g., *uivot, reistoa, sohdi*)	
7) Word recognition (ALLU, TL2 A; 5 min; matching word to pictures)	Test
**Spelling**	
1) Letters (29 letters)	Test
2) Content words (4 words; *koti, kirja, joulu, isä*)	
3) Syllables (9; e.g., *is, vor, ke*)	
4) Bi-syllabic nonwords (9; e.g., *ysö, vami, evot*)	
5) Complex nonwords (9; e.g., *hoipi, onkula*)	
**Mathematics**	
1) Numbers (6 tasks)	Test
4) Comparison of auditorily presented numbers	
5) Identification of auditorily presented numbers	
6) Counting (Adots@)	
**Rapid naming**	
**Control measures: Simple reaction time, articulation time, touch reaction**	Cognitive Workshop
**Interview of the child**	Interview by the tester
1) Achievement strategies (17 items)	
2) General attributions (4 items: success/failure)	
3) Task-specific attributions x 2 (effort, skill, difficulty level)	
**Ratings**	
1) Child=s strategies (e.g., task avoidance, persistence)	Ratings by the tester
2) Task attention	
**Parent questionnaire**	
1) Spelling skills	Parental report
2) Reading skills	
3) Timing of reading acquisition	
4) Attributions of success/failure, beliefs about child=s achievement, child=s strategies in achievement situations	

GRADE 1: December, Group assessments at school	Data
**Spelling**<br>1) Syllables *(9; eh, vis, li)*<br>2) Bi-syllabic nonwords *(9; värö, ame, hopa)*<br>3) Complex nonwords *(9; hoipi, onkula, uivot)*<br>4) Error searching: AVekku's Spelling Errors@ (6 min; *sadi, maato, sikä*)	Test
**Reading**<br>1) Word recognition (ALLU, TL2; matching word to pictures)	Test<br>Test
**Teacher ratings**<br>1) Reading (1 - 5; 1 = severe difficulties; 5 = very good skills)<br>2) Spelling (1 - 5; 1 = severe difficulties; 5 = very good skills)	Ratings by the teacher
**Teacher reports**<br>1) Letters taught in the classroom by December<br>2) Type of reading difficulties<br>3) Type of spelling difficulties<br>4) More detailed description of reading problems (onset, remediation)<br>5) Description of other learning difficulties<br>6) Timing of reading acquisition (grasping of the principle of assembly)	Reports by the classroom teacher
**Special education teacher reports**<br>3) Classroom: Number of pupils in remediation & their problems<br>4) Types of reading & spelling difficulties<br>5) JLD child: Types of other learning difficulties & remediation	Reports by the special education teacher
**Parent questionnaire**<br>1) Spelling skills<br>2) Reading skills (+ in Dec reading other materials than home work)<br>3) Timing of reading acquisition<br>4) Attributions of success/failure, beliefs about child=s achievement, child=s strategies in achievement situations	Parental report

GRADE 1: January-March, Individual assessments	Data
NEPSY – Neuropsychological Assessment (8 subtests)<br>*Attention/Executive:* Tower, Knock and tap, Visual attention. *Language:* Comprehension of instructions, Oromotor sequences. *Sensorimotor:* Fingertip tapping, *Memory:* Memory for names (immediate & delayed), Sentence repetition	Test
VMI (Developmental Test of Visual-motor Integration; 24 items)	Test
Morphology Test (30 items)	Test
Rapid naming (RAN): Objects, letters, numbers, object names	Test
**Phonological awareness**<br>1) Initial phoneme production & deletion (*asuu* ∃ *suu*)<br>2) Non-word repetition (18 items; *sipa, kutta, tiippa, tati, kuuki* ...)	Test<br>Cognitive Workshop
**Reading**<br>1) Letter naming (29 letters)<br>2) Syllables (9; *is, vor, ke*)<br>3) Bi-syllabic nonwords (9; *ysö, vami, evot*)<br>4) Complex nonwords (9; *päyhä, onsura, eivot*)	Test
Auditory modulation detection, "Parrot" (by John Stein)	Computer test

GRADE 1: April, Group assessments at school	Data
**Spelling**	
1) Syllables (9; *eh, vis, li*)	Test
2) Bi-syllabic nonwords (9; *värö, ame, hopa*)	Test
3) Complex nonwords (9; *hoipa, oipus, ankula*)	Test
4) Error searching: AVekku's Spelling errors; 6 min; *jona, kaala, uido* ...)	
**Reading**	
1) Word recognition (ALLU, TL2B; 5 min; matching word to pictures)	Test
2) Reading comprehension: "Vekku's Stories" (4 sentences/picture, "find the word in one of the sentences which does not match the picture")	Test<br>Test
**Teacher ratings**	
1) Reading (1 - 5; 1 = severe difficulties; 5 = very good skills)	Ratings by the teacher
2) Spelling (1 - 5; 1 = severe difficulties; 5 = very good skills)	
**Teacher reports**	
1) Type of reading difficulties	Reports by the classroom teacher
2) Type of spelling difficulties	
3) More detailed description of reading problems (onset, remediation)	
4) Description of other learning difficulties	
5) Timing of reading acquisition (grasping of the principle of assembly)	
6) Motivation	
**Special education teacher reports**	Reports by the special education teacher
1) Classroom: Number of pupils in remediation & their problems	
2) JLD child: Reading & spelling difficulties, types of other learning difficulties & remediation	

GRADE 1: May, Individual assessment	Data
**Phonological awareness**	
1) Phoneme blending (Poskiparta ym.; 10 items; *p-u-u*)	Test
2) Syllable deletion (Poskiparta ym.; 10 items; *paluu* ∃ *luu*)	Test
3) Initial phoneme production & deletion (Poskiparta ym.; 10 items; *asuu* ∃ *suu*)	Test
4) Phonological Common Unit Task: Syllable & phoneme level (*lehmä – lehti; noita - kukka*)	CW
**Phonological processing**	
1) Perception, "Space alien" –task (22 items; "same or different")	CW
2) Production (19 items; *kanspultine, lipikuta, meppotto, naukutakoo*)	CW
3) Spelling (3 words; äiti, koulu, isä; 17 nonwords; *simpuu, rameli, trooki*)	Scriptlog
**Reading**	
1) Letter naming (29 letters)	Cognitive Workshop
2) Content words (9 words; *koti, juosta, kolme, kirja*)	
3) Function words (9 words; *siellä, se, vain, mikä*)	
4) Syllables (9; *eh, vis, li, nä, kup*)	
5) Bi-syllabic nonwords (9; *värö, ame, hopa, ypöt*)	
6) Complex nonwords (9; *uivot, reistoa, sohdi, hoipi*)	
7) Oral text reading, "Exiting adventures", minimum duration 3 minutes: a) fluency (words/minute) & accuracy (b) comprehension (3 questions)	Test audiotaping

GRADE 1: May, Individual assessment (continued)	Data
**<u>Spelling</u>**<br>1) Letters (29 letters)<br>2) Syllables (9 items; *is, vor, ke, tyn, ju*)<br>3) Bi-syllabic nonwords (9 items; *ysö, vami, evot*)<br>4) Complex nonwords (9 items; *hoipi, onkula, uivot, äystä*)	 Test<br>Test<br>Test<br>Test
**<u>Mathematics</u>**<br>1) Numbers: (6 tasks)<br>2) Comparison of auditorily presented numbers (12; "Which number is bigger")<br>3) Identification of aud. presented numbers (12; "Circle the number that you heard")<br>4) Counting (6 items; Adots@)<br>5) Arithmetics (12 items; *4 + 5, 8 – 3, 527 + 31, 485 - 42*)	 Test<br>Test<br>Test<br>Test<br>Test
**<u>Control measures</u>: 1) Simple reaction time (V è "nyt"), 2) Articulation time ("pupu")**	Cognitive Workshop
**<u>Interview of the child</u> (April or May)**<br>1) Achievement strategies (20 items; e.g., *I delay beginning tasks, I try hard to solve even the more difficult tasks*)<br>2) General attributions (4 items: success/failure; knows/does not know)<br>3) Task-specific attributions x 2 (2 items: effort, skill, difficulty level)<br>4) Motivation in school subjects (8 items, scale 1 - 5*; reading, spelling, maths, crafts, physical education, arts, music, reading at home*)	 Child=s interview by the tester
**<u>Ratings</u>**<br>1) Child=s strategies: active task orientation, active task avoidance, passivity, dependence on the adult, anxiety (10 items, scale 1 – 7; *When running into difficulty the child turns to the tester*)<br>2) Attention, hyperactivity, impulsiveness (19 items, scale 1 – 7)	Ratings by the tester
**<u>Parent questionnaires</u>**<br>1) Spelling<br>2) Reading<br>3) Timing of reading acquisition<br>4) Child's interest in reading<br>5) Home work<br>6) Attributions of success/failure, beliefs about child=s achievement, child=s strategies in achievement situations	 Parental report

8-year questionnaires	
**<u>Parent questionnaires</u>**<br>1) Reading, joint activities with the child, hobbies, use of the computer<br>3) Behavior Assessment System for Children (BASC)<br>2) Parenting attitudes<br>3) Parental achievement strategies & self-esteem<br>4) Family status & employment,<br>5) Stress, social support, work satisfaction	Parental report

GRADE 2: November, Group assessments at school	Data
**<u>Spelling</u>**	
**1) Nonwords 1 (9 items*; hoipa, oipus, ankula)***	**Test**
**2) Nonwords 2 (9 items ; *lirestemmä, märskynäivä, syönikeitväs*)**	
**3) Error searching; "Vekku's Spelling Errors" (2 min; *suusi, tytö, orave*)**	
**<u>Reading</u>**	
**1) Word recognition: ALLU, TL2 (2 min; matching word to picture)**	**Test**
**2) Sentence recognition: ALLU, TL4 (2 min; matching sentence to picture)**	
**3) Judgment of claims: ALLU, TL3 (5 min; *People eat books* - true/false**	
**4) Segmentation of sentences into words: Word Chains Test (1 min 25 sec)**	
**5) Judgment of words:Word Chains Test (50 sec; *meri, seri* - word/nonword)**	
**6) Segmentation of words into syllables: Word Chains Test (1 min 15 sec)**	
**7) Reading comprehension: Vekku's Stories (12 items, 4 sentences/picture; "find the word in one of the sentences which does not match the picture")**	
**8) Reading comprehension: ALLU, LY2 "Judo"(45 min; Child responds to 12 multiple choice questions after reading the text)**	
**<u>Mathematics</u> (from NMART, Räsänen, 2000)**	**Test**
**1) Arithmetic reasoning: "Number boxes" (10 min; "fill in the the missing number in the number series")**	
**2) Visual search of target numbers: a) number 5, b) numbers between 45 and 55 ("underline the targets as fast as you can")**	
**<u>Teacher ratings</u>**	
**1) Reading (scale 1 - 5; 1 = severe difficulties; 5 = very good skills)**	**Ratings by the teacher**
**2) Spelling (scale 1 - 5; 1 = severe difficulties; 5 = very good skills)**	
**<u>Teacher reports</u>**	
**3) Type of reading difficulties**	**Reports by the classroom teacher**
**4) Type of spelling difficulties**	
**5) More detailed description of reading problems (onset, remediation)**	
**6) Description of other learning difficulties**	
**<u>Special education teacher reports</u>**	**Reports by the special education teacher**
**1) Classroom: Number of pupils in remediation & their problems**	
**2) Types of reading & spelling difficulties**	
**3) JLD child: Types of other learning difficulties & remediation**	
**<u>Parent questionnaire</u>**	
**1) Spelling**	**Parental report**
**2) Reading**	
**3) Timing of reading acquisition**	
**4) Attributions of success/failure, beliefs about child=s achievement, child=s strategies in achievement situations**	

**GRADE 2: October/November, Individual assessments**	**Data**
**WISC III; Wechsler Intelligence Scale for Children (*Wechsler, 1991*) – short form (5 verbal + 4 performance scale subtests)**<br>**1) Vocabulary**<br>**2) Arithmetic**<br>**3) Comprehension**<br>**4) Similarities**<br>**5) Digit Span**<br>**6) Picture Completion**<br>**7) Block Design**<br>**8) Object Assembly**<br>**9) Coding**	**Test**
**<u>Parent-child interaction</u>**<br>**1) Card arrangement task: Part 1, One-way communication, Part 2, Two-way communication *(Ditton, Green & Singer, 1987; Rasku-Puttonen, Lyytinen, Poikkeus, Laakso & Ahonen, 1994)*: Coding: parental communication clarity, quality of instructions**<br>**2) Word learning, 10 minutes: Mother is asked to help her child to learn pseudoword names for f 5 pictured objects (auto = rytvänä, pallo = moini, omena = lumpi, talo = töiterö, pipo = purmu). Coding: parental teaching strategies, quality of interaction**	**Videotape**
**<u>Motor skills: Balancing</u>**<br>**1) Static balance: The child stands still on a balance recording device for 30 second in 5 conditions (eyes open & gaze fixed on a target or eyes closed, feet apart or feet together or one foot ahead of the other)**<br>**2) Dynamic balance: The child stands on a balance recording device and with slight body movement makes the cursor move from one target to next on the monitor's screen**	
**GRADE 2: April, Group assessments at school**	**Data**
**<u>Spelling</u>**<br>**1) Nonwords 1 (4*; moivura, sehdi, insot, rölskä)***<br>**2) Nonwords 2 (9; *syönitiippa, kähynäivä, rynistemmä, kenstileisas*)**<br>**3) Error searching: "Vekku's Spelling Errors" (2 min; *jona, kaala, uido*)**	**Test**
**<u>Reading</u>**<br>**1) Word recognition (ALLU, TL2; matching word to picture)**<br>**2) Segmentation of sentences into words: Word Chains Test**<br>**3) Reading comprehension: Vekku's Stories (*Ressu karkaa, Isä poistaa tahran, Mustetarha tuolissa, Sulamaton lumiukko*; 12 pictures; 4 sentences/picture, 10 min; "find the word in one of the sentences which does not match the picture")**<br>**4) Reading comprehension: ALLU, LY2, "Morning exercises" (45 min; Child responds to multiple choice questions after reading the text)**	**Test**
**Arithmetic reasoning (from NMART, Räsänen, 2000, orig. Langdon & Warrington): Child is asked to continue number series by circling one of the alternative numbers (e.g., "What comes next in the series 5 10 15 – number 1, 25, 20 or 18?")**	**Test**
**<u>Teacher ratings</u>**<br>**1) Reading (1 - 5; 1 = severe difficulties; 5 = very good skills)**<br>**2) Spelling (1 - 5; 1 = severe difficulties; 5 = very good skills)**	**Ratings by the teacher**
**9-year questionnaires**	
**<u>Parent questionnaires</u>**<br>**1) Reading, joint activities with the child, hobbies, use of the computer**<br>**2) *Behavior Assessment System for Children (BASC)***<br>**3) Parenting attitudes**<br>**4) Family status & employment,**<br>**5) Mood, stress, social support**	**Parental report**

GRADE 2: June, Individual assessment	Data
**Reading**	
1) 3-syllabic words (10 items; *sininen, kiivetä, marssija*)	CW
2) 3-syllabic nonwords (10 items; *viepere, lunkkape, larvaanto*)	CW
3) 4-syllabic words (10 items; *koululainen, vaatimaton, kissanpentu*)	CW
4) 4-syllabic nonwords (10 items; *köylämönsi, hiemakkola, tullakangit*)	CW
5) List reading of words: Lukilasse (Häyrinen, Serenius-Sirve & Korkman; 2 min, 90 words; *eli, soi, jäät, kangerrella, kansanedustaja, snorkkelisukeltaja*). Accuracy & Speed.	Test
6) Oral text reading, "Exiting adventures", minimum duration 3 minutes: a) fluency (words/minute) & accuracy (*Antin isoisä oli merikapteeni*)	Test audiotaping
7) Oral nonword text reading, "Vinnittäjiä tenkoja": a) fluency (words/minute) & accuracy. 4 sentences (*Intän äsiäni ila remipentaani*).	Test audiotaping
**Spelling**	
8) 4-syllabic words (6 items; vahingossa, leivänpaahdin, lahjakortti)	Test
9) 4-syllabic nonwords, 1 additional repetition by child's request (6 items; *moinipuuli*)	Test
10) 4-syllabic nonwords (6 items; *peunumiile, mainokkeri, kimpuraali*)	Test
**Rapid naming**	
1) Serial, RAN: Objects (10 x 5 items; *auto, talo, kala, kynä, pallo*)	Test
2) Serial, RAS: Colors, numbers, letters (50 items)	Test
3) Individually presented pictures of objects (3 x 5 items; *auto, talo, kala, kynä, pallo*)	CW
**Phonological processing**: Perception, "Space alien" –task (22 items; "same or different")	CW
**Auditive processing**	
1) "Parrot Task" (John Stein, Oxford): a) FM, Frequency Modulation (*which of the words was more wavy*), b) AM, Amplitude Modulation (*... more wobbly*)	Computer
2) Audilex (Kai Karma): visuo-auditive perception (*press the bar when it is turn for the last note in the melody*)	Computer
3) "Dino Tasks" (Usha Goswami, Jenny Thomson): a) P-Centre Task (*show me which of the two dinos has a stronger rhythm in his sound*), b) Rise Time Task (*show me which dino sounds different from the other dinos*)	Computer
**Mathematics**	
1) Numbers: (6 tasks)	Test
2) Arithmetics (5 min; *11 – 2, 22 + 13 – 8, 3 . 7 + 12*)	Test
**Interview of the child** (April or May)	
1) Achievement strategies (20 items; e.g., *I delay beginning tasks, I try hard to solve even the more difficult tasks*)<br>2) General attributions (4 items: success/failure; knows/does not know)<br>3) Task-specific attributions x 2 (2 items: effort, skill, difficulty level)<br>4) Motivation in school subjects (10 items, scale 1 - 5*; reading, spelling, maths, crafts, physical education, arts, music, religion, biology, reading at home*)	Child=s interview by the tester
**Ratings**	Ratings by the tester
1) Child=s strategies: active task orientation, active task avoidance, passivity, dependence on the adult, anxiety (10 items, scale 1 – 7; *When running into difficulty the child turns to the tester*)	
2) Attention, hyperactivity, impulsiveness (19 items, scale 1 – 7)	
**Parent questionnaires**	
• Spelling & Reading, Child's interest in reading<br>• Home work<br>• Attributions of success/failure, beliefs about child=s achievement, child=s strategies in achievement situations	Parental report

GRADE 3: March, Group assessments at school	Data
**Spelling**	
1) Nonwords 1 (10; rinti, etti, ruumpii)	Test
2) Nonwords 2 (10; keinerä, dukkimaas, etakkajuut)	
3) Writing a story from pictures:5 different pictures	
**Reading**	
5) Word recognition (ALLU, TL2; matching word to picture)	Test
6) Segmentation of sentences into words: (ALLU TL5)	
7) Reading comprehension: ALLU, LY3, "Camera instrcutions" (45 min; Child responds to multiple choice questions after reading the text)	
8) Reading comprehension, mark the unfitting words, "The plants' need of light"	
**Morphology**	
1) Test of inflectional morphology (Written responses, inflection with non words)	Test
Arithmetic reasoning (from NMART, Räsänen, 2000, orig. Langdon & Warrington): Child is asked to continue number series by circling one of the alternative numbers (e.g., "What comes next in the series 5 10 15 – number 1, 25, 20 or 18?")	Test
**Teacher ratings**	
3) Reading (1 – 5; 1 = severe difficulties; 5 = very good skills)	Ratings by the teacher
4) Spelling (1 – 5; 1 = severe difficulties; 5 = very good skills)	

GRADE 3: April, Individual assessment	Data
**Reading**	
1) 3-syllabic nonwords (10 items; *viepere, lunkkape, larvaanto*)	CW
2) 4-syllabic nonwords (10 items; *köylämönsi, hiemakkola, tullakangit*)	CW
3) Rapidly presented words with mask (18 items; *on, se, opettaja, kuningas)*	CW
4) List reading of words: Lukilasse (Häyrinen, Serenius-Sirve & Korkman; 2 min, 105 words; *eli, soi, jäät, kangerrella, kansanedustaja, snorkkelisukeltaja*). Accuracy & Speed.	Test
5) Oral text reading, "Nonsense things", minimum duration 3 minutes: a) fluency (words/minute) & accuracy (*Amerikassahan on kuulemma kaikkea*)	Test audiotaping
6) Oral nonword text reading, "Vinnittäjiä tenkoja": a) fluency (words/minute) & accuracy. 8 sentences (*Intän äsiäni ila remipentaani*).	Test audiotaping
**Spelling**	
1) 4-syllabic words (10 items; professori, olympiastadion, trigonometria)	Test
2) 4-syllabic nonwords, (12 items; *moinipuuli, paunitteri, lantureeli*)	Test
3) 2-syllabic nonwords (12 items; *sinti, sintti, sintuu, sinttuu*)	Scriptlog
**Rapid naming**	
1) Serial, RAN: Objects (10 x 5 items; *auto, talo, kala, kynä, pallo*)	Test
2) Serial, RAN: Letters (10 x 5 items; *O, A, S, T, P*)	Test
3) Serial, RAN: Numbers (10 x 5 items; *2, 6, 9, 4, 7*)	Test
4) Individually presented pictures of objects (3 x 5 items; *auto, talo, kala, kynä, pallo*)	Test
**Phonological processing**:	
1) Perception, "Space alien" –task (22 items; "same or different")	CW
2) Phonological Common Unit Task: Phoneme level (*lauhkua-terike upittaa- homppe*)	CW
**Auditive processing**	

1) "Parrot Task" (John Stein, Oxford): a) FM, Frequency Modulation (*which of the words was more wavy*), b) AM, Amplitude Modulation (... *more wobbly*)	Computer
<u>Mathematics</u>	
1) Numbers: (6 tasks)	Test
2) Arithmetics (5 min; *11 – 2, 22 + 13 – 8, 2*8*)	Test
<u>Memory</u>	Test
1) Digit Span	
<u>Interview of the child</u> (April or May)	
1) Achievement strategies (20 items; e.g., *I delay beginning tasks, I try hard to solve even the more difficult tasks*)	Child=s interview by the tester
2) General attributions (4 items: success/failure; knows/does not know)	
3) Task-specific attributions x 2 (2 items: effort, skill, difficulty level)	
4) Motivation in school subjects (10 items, scale 1 - 5*; reading, spelling, maths, crafts, physical education, arts, music, religion, biology, reading at home*)	
<u>Ratings</u>	Ratings by the tester
1) Child=s strategies: active task orientation, active task avoidance, passivity, dependence on the adult, anxiety (10 items, scale 1 – 7; *When running into difficulty the child turns to the tester*)	
2) Attention, hyperactivity, impulsiveness (19 items, scale 1 – 7)	
<u>Parent questionnaires</u>	
• Spelling & Reading, Child's interest in reading	Parental report
• Home work	
• Attributions of success/failure, beliefs about child=s achievement, child=s strategies in achievement situations	
**GRADE 5: April, Individual assessment**	**Data**
<u>Reading</u>	
1) List reading of words: Lukilasse (Häyrinen, Serenius-Sirve & Korkman; 1 min, 105 words; *eli, soi, jäät, kangerrella, kansanedustaja, snorkkelisukeltaja*). Accuracy & Speed.	Test
<u>Spelling</u>	
1) 2-syllabic nonwords (24 items; *siinttu, siinttuu,senkkuu, seenkkuu*)	Scriptlog
<u>Self report</u>	
• Achievement strategies (20 items; e.g., *I delay beginning tasks, I try hard to solve even the more difficult tasks*)	Self report
• Motivation in school subjects (10 items, scale 1 - 5*; reading, spelling, maths, crafts, physical education, arts, music, religion, biology, reading at home*)	
<u>Parent questionnaires</u>	
• Spelling & Reading, Child's interest in reading, Time spent to homework	Parental report
• Attributions of success/failure, beliefs about child=s achievement	
• Child's motor skills	
<u>Teacher questionnaire</u>	
• Reading, spelling, math skills	Teacher report
• Motor skills	
• Need of special support in learning	
**GRADE 7: February-March, Group assessments at school**	**Data**
<u>Reading</u>	
1) Word segmentation, *1 min 30s*, Nevala	Test
2) Searching errors in spelled words, *3 min. 30s* Nevala	Test
3) Reading comprehension: underline the inappropriate words in the text	Test
4) Salzburg, reading fluency –test, *3 min*	

**<u>Mathematics</u>**	
**KTLT-A, arithmetic tasks which are presented in written form to the child. One third of the items is multiple choice task and rest are open ended. (Räsänen, P. & Leino, L., Niilo Mäki Instituutti (2005))**	**Test**
**<u>Arithmetic reasoning</u>**	**Test**
**Child's task is to reason out the missing number. Reasoning is based on the logic of other presented numbers in the 3*3 matrix (from NMART, Räsänen, P., Niilo Mäki Instituutti)**	
**<u>Self report</u>**	
• **Reading habits and use of computer**	**Self report**
**<u>Parent questionnaires</u>**	
**Concerning child:**	**Parental report**
• **Reading habits and use of computer**	
• **Reading, spelling, math skills**	
• **Homework**	
• **Parenting**	
• **Attributions of success/failure, beliefs about child=s achievement**	
• **Strengths and weaknesses**	
**Concerning self:**	
• **Family structure**	
• **Education, employment**	
• **Stress and mood**	
**GRADE 8: September-November, Individual assessments at the university**	**Data**
**<u>Reading</u>**	
**1) List reading: Words (Lukilasse), 1 min time limit, max 105 words**	**Test**
**2) Text reading: "Tunturilappi" Part 1, aloud**	**CW**
**3) Text reading: "Tunturilappi" Part 2 Silent reading, marking unfit words**	
**4) Single word reading, Rapidly presented words (100 ms), 18 items**	**CW**
**5) Non word text reading – "Vinnittäjiä tenkoja"**	**CW**
**<u>Spelling</u>**	
**1. Words, 10 items**	**CW**
**2. Non words with 3-4 syllables, 20 items**	**CW**
**3. Non word text in sentences, 6 sentences**	**CW**
**4. Non words with Scriptlog, 12 items**	**Scriptlog**
**<u>Rapid Naming, RAN</u>**	
**1. Objects**	**Test**
**2. Letters**	**Test**
**3. RAS: Letters + numbers + colors**	**Test**
**<u>Auditory perception</u>**	
**1. Duration perception, 22 items**	**CW**
**2. Eke-Ekke categorization, 80 items**	**CW**
**3. Y – I – non speech, 80 items**	**CW**
**4. Y – I – speech, 80 items**	**CW**
**<u>Phonology</u>**	
**1. Phoneme deletion, 20 items**	**CW**
**2. Common unit – Nonwords, 15 items**	**CW**
**<u>Memomry</u>**	
**1. Digit Span Numbers Forwards and Backwards**	**Test**
**2. Digit Span syllables Forwards and Backwards**	**Test**

<u>Self report</u><br>**Strengths & Difficulties Questionnaires, SDQ,** Goodman R (1997) The Strengths and Difficulties Questionnaire: A Research Note. *Journal of Child Psychology and Psychiatry*, 38, 581-586.	**Self report**
<u>Parent report</u><br>**Strengths & Difficulties Questionnaires, SDQ,** Goodman R (1997) The Strengths and Difficulties Questionnaire: A Research Note. *Journal of Child Psychology and Psychiatry*, 38, 581-586.	**Parent report**
**GRADE 9: March-April, Group assessments at school**	**Data**
**<u>Reading</u>**<br>**1) Word segmentation, *1 min 30s,* Nevala**<br>**2) Searching errors in spelled words, *3 min.* Nevala**<br>**3) Reading comprehension: underline the inappropriate words in the text**<br>**4) Salzburg, reading fluency –test, 2 *min***	 **Test**<br>**Test**<br>**Test**
**<u>Mathematics</u>**<br>**KTLT-A, arithmetic tasks which are presented in written form to the child. One third of the items is multiple choice task and rest are open ended. (Räsänen, P. & Leino, L., Niilo Mäki Instituutti (2005))**	 **Test**
**<u>Arithmetic reasoning</u>**<br>**Child's task is to reason out the missing number. Reasoning is based on the logic of other presented numbers in the 3*3 matrix (from NMART, Räsänen, P., Niilo Mäki Instituutti)**	 **Test**
**<u>Self report</u>**<br>• **Reading habits and use of computer**<br>**<u>Parent questionnaires</u>**<br>**Concerning child:**<br>• **Reading habits and use of computer**<br>• **Reading, spelling, math skills**<br>• **Homework**<br>• **Parenting**<br>• **Attributions of success/failure, beliefs about child=s achievement**<br>• **Strengths and weaknesses**<br>**Concerning self:**<br>• **Family structure**<br>• **Education, employment**<br>• **Stress and mood**	 **Self report**<br><br>**Parental report**
