# Supplementary material for: Unveiling the Mysteries of Dyslexia—Lessons Learned from the Prospective Jyväskylä Longitudinal Study of Dyslexia
Source: Brain Sci. 2021 Mar 27;11(4):427. doi: 10.3390/brainsci11040427 (PMC8066413; doi:10.3390/brainsci11040427)
Supplement: Supplementary file 1 [file brainsci-11-00427-s001.zip › Table S2_Summary of the technical details in EEG measurements.pdf]

TABLE S2 Summary of the technical details in EEG measurements.

	Newborns	6 months & 6.5 years	Grade 3
No. of electrodes	6 Ag/AgCl	10 Ag/AgCl	128 Ag/AgCl
Reference	Ipsilateral mastoids	Ipsilateral mastoids	Cz
Sampling rate	200 Hz	200 Hz	500 Hz
Online filtering	0.5–35 Hz	0.5–35 Hz	0.1–100 Hz
Offline filtering	-	-	0.53–35 Hz, 50 Hz
Artefact criteria	> ±200 μV	> ±200 μV	> ±200 μV

Note. Ag/AgCl, silver/silver chloride electrode.
